# Bioactive Patch for Rotator Cuff Repairing via Enhancing Tendon‐to‐Bone Healing: A Large Animal Study and Short‐Term Outcome of a Clinical Trial

**DOI:** 10.1002/advs.202308443

**Published:** 2024-06-23

**Authors:** Yuhao Kang, Liren Wang, Shihao Zhang, Bowen Liu, Haihan Gao, Haocheng Jin, Lan Xiao, Guoyang Zhang, Yulin Li, Jia Jiang, Jinzhong Zhao

**Affiliations:** ^1^ Department of Sports Medicine Department of Orthopedics Shanghai Institute of Microsurgery on Extremities Shanghai Sixth People's Hospital Affiliated to Shanghai Jiao Tong University School of Medicine No. 600 Yishan Road Shanghai 200233 China; ^2^ Regenerative Sports Medicine and Translational Youth Science and Technology Innovation Workroom Shanghai Jiao Tong University School of Medicine No. 600 Yishan Road Shanghai 200233 China; ^3^ Key Laboratory for Ultrafine Materials of Ministry of Education Frontiers Science Center for Materiobiology and Dynamic Chemistry Engineering Research Center for Biomedical Materials of Ministry of Education School of Materials Science and Engineering East China University of Science and Technology Shanghai 200237 China; ^4^ Bioarticure Medical Technology (Shanghai) Co., Ltd No.81‐82, Zuchongzhi Road, Pudong Shanghai 200120 China; ^5^ School of Mechanical, Medical and Process Engineering Center of Biomedical Technology Queensland University of Technology Brisbane 4059 Australia

**Keywords:** rotator cuff tear, tendon–bone interface, umbilical cord

## Abstract

Tissue engineering has demonstrated its efficacy in promoting tissue regeneration, and extensive research has explored its application in rotator cuff (RC) tears. However, there remains a paucity of research translating from bench to clinic. A key challenge in RC repair is the healing of tendon–bone interface (TBI), for which bioactive materials suitable for interface repair are still lacking. The umbilical cord (UC), which serves as a vital repository of bioactive components in nature, is emerging as an important source of tissue engineering materials. A minimally manipulated approach is used to fabricate UC scaffolds that retain a wealth of bioactive components and cytokines. The scaffold demonstrates the ability to modulate the TBI healing microenvironment by facilitating cell proliferation, migration, suppressing inflammation, and inducing chondrogenic differentiation. This foundation sets the stage for in vivo validation and clinical translation. Following implantation of UC scaffolds in the canine model, comprehensive assessments, including MRI and histological analysis confirm their efficacy in inducing TBI reconstruction. Encouraging short‐term clinical results further suggest the ability of UC scaffolds to effectively enhance RC repair. This investigation explores the mechanisms underlying the promotion of TBI repair by UC scaffolds, providing key insights for clinical application and translational research.

## Graphical abstract

Tissue engineering for rotator cuff (RC) tears is promising but lacks clinical translation. Umbilical cord (UC) rich in bioactive elements show potential for tendon–bone interfaces (TBI) healing. UC scaffolds improve cell function, chondrogenesis, and anti‐inflammation. Canine RC repair model and clinical results confirm their effectiveness in TBI repair. This study delves into UC scaffold mechanisms, offering insights for clinical application and translational research.

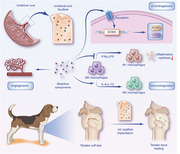

## 1 Introduction

Rotator cuff (RC) tear is a highly prevalent disease of the shoulder, often characterized by shoulder pain and limited range of motion. Its prevalence in the population ranges from ≈15% to 51%, with the likelihood of incidence increasing with age.^[^ ^1^ ^]^ For ameliorating symptoms of RC tear, surgical repair remains one of the foremost approaches. Nevertheless, it is still not entirely successful, due primarily to difficulties in the healing process at the tendon–bone interface (TBI).^[^ ^2^ ^]^ The tendon–bone enthesis region consists of four continuous zones: tendon, uncalcified fibrocartilage, calcified fibrocartilage, and bone.^[^ ^3^ ^]^ Following RC repair, the tendon remanent and bone are directly connected by scar tissue instead of natural fibrocartilage, which lacks the ability to disperse concentrated stresses, leading to the recurrence of RC tears.^[^ ^4^ ^]^ Reconstructing the structure of TBI and restoring its mechanical properties is the key issue that requires urgent attention and resolution.

Surgical repair with graft or patch augmentation is commonly performed to enhance the mechanics of RC tissue. Currently, clinically approved materials can be classified into xenografts, synthetic materials, and allografts. Xenografts, even after decellularization, still elicit immune reactions leading to complications.^[^ ^5^ ^]^ While synthetic materials possess low immunogenicity and high strength, their degradation byproducts may still trigger immune rejection and hinder tissue repair, resulting in a high retear rate (10–62%).^[^ ^6^ ^]^ Allografts, such as decellularized human dermal patches, have lower strength compared to synthetic grafts but exhibit good biocompatibility, effectively increase tendon thickness after repair, and potentially enhance the mechanical strength of the repaired tissue. However, they have limited biological activity and still carry a certain failure rate. Therefore, it is crucial to develop tissue‐engineered scaffolds containing bioactive elements with regenerative abilities.

Commercially available products are mainly used on the surface of repair sites to enhance tissue thickness. In studies, tissue engineering materials often serve as bridging structures to connect tendons and bones, such as decellularized tendon–bone composites,^[^ ^7^ ^]^ decellularized tendons,^[^ ^8^ ^]^ and decellularized extracellular matrix (ECM).^[^ ^9^ ^]^ In recent years, more focus has been placed on the implants situated between the tendon and bone at TBI, aiding in cell adhesion and proliferation, as well as dispensing bioactive components to enhance the mechanical strength of the TBI and promote tendon–bone integration. Although some studies have validated these approaches in large animal experiments, there have been no clinical reports to date.^[^ ^10^ ^]^

The umbilical cord (UC) serves as an important biobank of cells and bioactive substances, including various cytokines, ECM components, and numerous mesenchymal stem cells (MSCs). In recent years, it has gained increasing attention due to its availability as medical waste and its harmlessness to donors.^[^ ^11^ ^]^ The UC or UC‐related tissues have multiple applications in sports medicine, such as tendon repair,^[^ ^12^ ^]^ cartilage repair,^[^ ^13^ ^]^ and RC repair.^[^ ^14^, ^15^ ^]^ The application of decellularized UC scaffolds for rabbit RC injuries was initially reported by Yuan et al. However, it is widely recognized that small and large animals have different healing capabilities. In small animal models, untreated injuries can sometimes heal on their own. To better mimic the potential use of UC scaffolds in clinical settings, we developed a canine RC tear model, specifically created to support the translation of UC scaffolds from bench to clinical.

**Figure 1:**
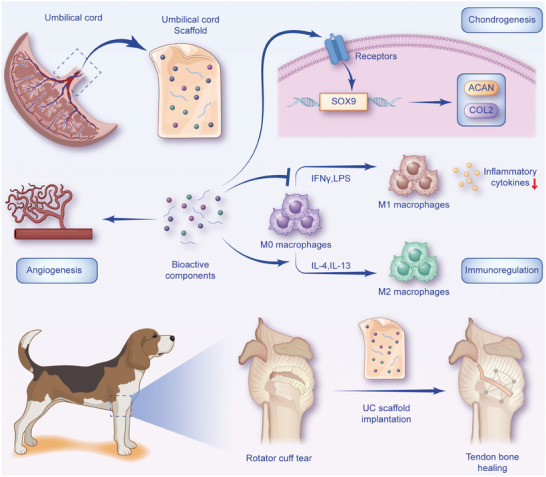
Schemata of UC scaffold enhancing tendon–bone interface healing. UC, umbilical cord.

In accordance with the relevant regulations of the U.S. Food and Drug Administration (FDA) pertaining to human cells, tissues, and cellular and tissue‐based products (HCT/Ps) and preserving most of the bioactive components in UC,^[^ ^16^ ^]^ we subjected human UC tissue to minimal manipulating to produce a new biomaterial scaffold for promoting TBI regeneration. In vitro experiments demonstrated that the extracts from the UC scaffold exhibited anti‐inflammatory effects, and chondrogenic effects by influencing ECM synthesis metabolism. In the canine model of RC repair, implantation of the UC scaffold demonstrated favorable biologic therapeutic effects, verified by histology, biomechanics, and Magnetic resonance imaging (MRI). Finally, the short‐term outcome of a clinical trial demonstrated that UC scaffold implantation leads to early healing at TBI (**Figure** **1**).

## 2 Result

### 2.1 Characterization of UC Scaffold

**Figure 2:**
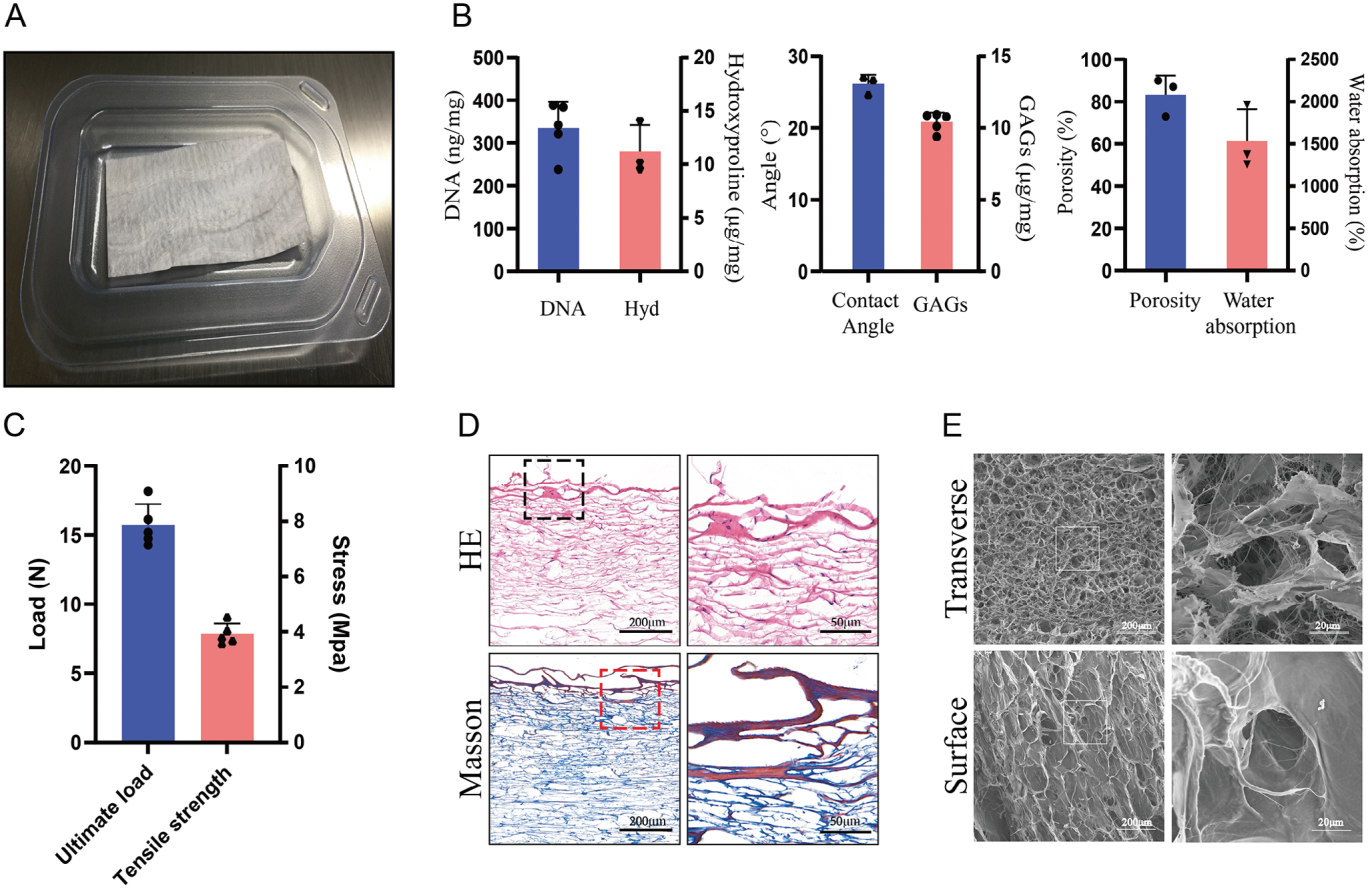
UC Scaffold Characterization. A) UC scaffold; B) DNA content, Hydroxyproline content (Hyd), Glycosaminoglycans content (GAGs), Contact angle, Porosity and Water absorption of UC scaffold; C) Mechanical properties of UC scaffold; D) HE & Masson staining of UC scaffold(Left:10x and Right: 40x); E) SEM scanning of UC scaffold microstructure.

The processed UC scaffold is shown in **Figure** **2**A. First, we conducted mass spectrometry analysis and found that it is rich in various bioactive components, including collagen, TGF‐β, periostin, fibronectin, and others. Among the top 30 proteins with the highest coverage, collagen VI and TGFBI were selected for sustained‐release evaluation, which suggested that they could be released for 28 days. (Figures S1 and S2, Supporting Information). The DNA content in the UC scaffolds was measured to be ≈334.8 ng mg^−1^ (dry weight), and the glycosaminoglycans  (GAGs) and hydroxyproline content were 10.4 ± 0.6 and 11.2 ± 2 µg mg^−1^, individually (Figure 2B). The porosity of the UC scaffolds was 79.3 ± 16.8%, and the water absorption rate was 1598.7 ± 834.3%, besides, high hydrophobicity was confirmed by small contact angle (26.1 ± 1.0°, Figure 2B; Figure S3, Supporting Information). The ultimate load of the UC scaffolds was 15.7 ± 1.4 N, and the tensile strength was 7.85 ± 0.68 MPa (other properties are provided in Table S2, Supporting Information). Histological staining with HE and Masson's trichrome staining revealed its porous structure and rich collagen content. Observations under high magnification revealed that, despite the UC scaffolds not being subjected to decellularization, it harbors a sparse cellular population (Figure 2D). Scanning electron microscopy (SEM) indicated a 3D porous structure on the surface and cross‐section, which facilitates cell growth and adhesion (Figure 2E).

### 2.2 UC Scaffold with High Biocompatibility

**Figure 3:**
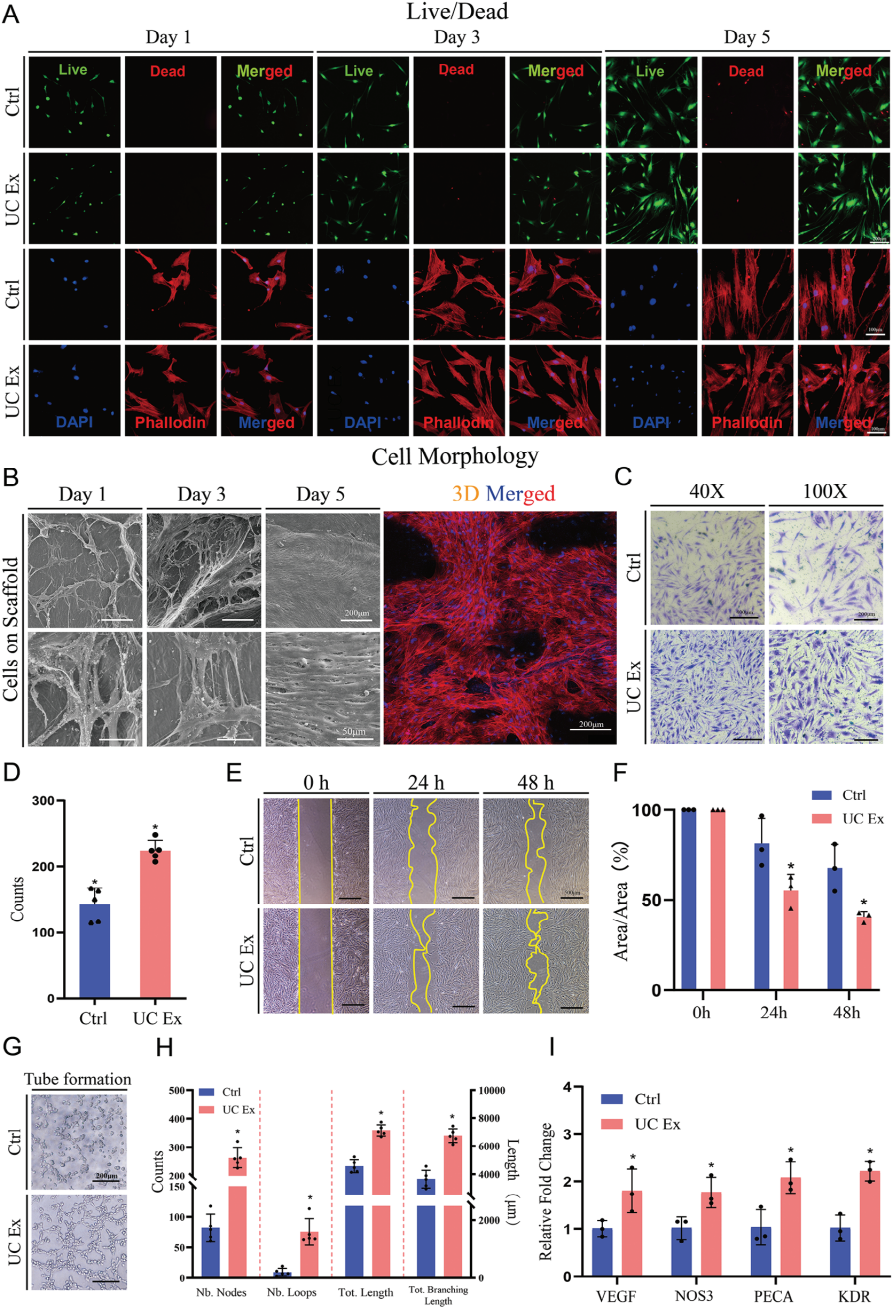
Biocompatibility of UC scaffold and effect of UC extracts on cell migration and angiogenesis. A) Live/dead staining and cell morphology cultured with UC extracts; B) BMSCs seeded on UC scaffold; C,D) Cell migration evaluation through transwell analysis; E,F) Cell migration evaluation through scratch test; G,H) Tube formation test cultured with UC extracts; I) Angiogenetic gene expression of HUVECs cultured with UC extracts. The results are presented as means ± SD. (^*^P < 0.05 compared with control).

The biocompatibility of the UC scaffold was assessed by culturing human bone marrow‐derived MSCs (BMSCs) in the UC extracts for 1, 3, and 5 days, then evaluating with the Cell Counting Kit‐8 (CCK‐8) and live/dead staining. The results of the live/dead cell staining suggested that the UC extracts did not affect cell viability. (**Figure** **3**A) The CCK‐8 results indicated that UC extracts promoted the proliferation of BMSCs, showing significant differences compared to the control group on the third and fifth days (P < 0.05) (Figure S4, Supporting Information). Fluorescence staining was performed to observe the morphology of BMSCs. The results indicated that there were no significant differences in the morphology of BMSCs cultured with UC extracts compared to the control group (Figure 3A). SEM scanning was performed on BMSCs cultured on the UC scaffold for 1, 3, and 5 days (Figure 3B). The results demonstrated favorable cell compatibility on the UC scaffold, with increasing coverage of the scaffold surface over time, exhibiting clustered and multilayered morphology, demonstrating the excellent cell compatibility of the UC scaffold. The aligned morphology of BMSC was observed at day 5, which is consistent with the fibrous topology and the directional cues inherent in the scaffold's structure. Confocal microscopy scanning revealed that BMSCs cultured on the scaffold for 5 days exhibited extensive surface coverage, indicating their proliferation on the scaffold (Figure 3B).

### 2.3 UC Scaffold Induces Cell Migration

We assessed the effects of UC extracts on BMSCs using scratch and transwell assays. In transwell assay, when UC extracts were added to the lower chamber, more cells were observed on the buttom of the chamber after 24 h of culture (P < 0.05), indicating that BMSCs were recruited by UC extracts (Figure 3C,D). The scratch assay showed that UC extracts could promote the migration of BMSCs, with significantly smaller scratch area after both 24 and 48 h compared to the control group (P < 0.05) (Figure 3E,F).

### 2.4 UC Scaffold Promotes Angiogenesis

Ibidi tube formation assay and RT‐qPCR analysis were conducted to evaluate the angiogenic effects of UC extracts on human umbilical vein endothelial cells (HUVECs). In tube formation assay, a greater number of nodes and loops is observed in the UC extracts group (P < 0.01), and both total length and total branching length are significantly higher than the control group (P < 0.01), suggesting that UC extracts have an angiogenic effect. (Figure 3G,H) Following 1 day of intervention with UC extracts, RT‐qPCR showed an upregulation of angiogenesis‐related genes, including VEGF, NOS3, PECA, and KDR, indicating that UC extracts have angiogenic effects (Figure 3I).

### 2.5 Anti‐Inflammation Evaluation

In order to investigate the immunomodulatory effects of UC extracts on macrophages, we evaluated their effects on M1 and M2 macrophage polarization using flow cytometry, immunofluorescence staining, reactive oxygen species (ROS) staining, RT‐qPCR, and inflammation cytokine arrays. In vivo, anti‐inflammation evaluation was performed in rats RC repair model (Figure S5, Supporting Information).

**Figure 4:**
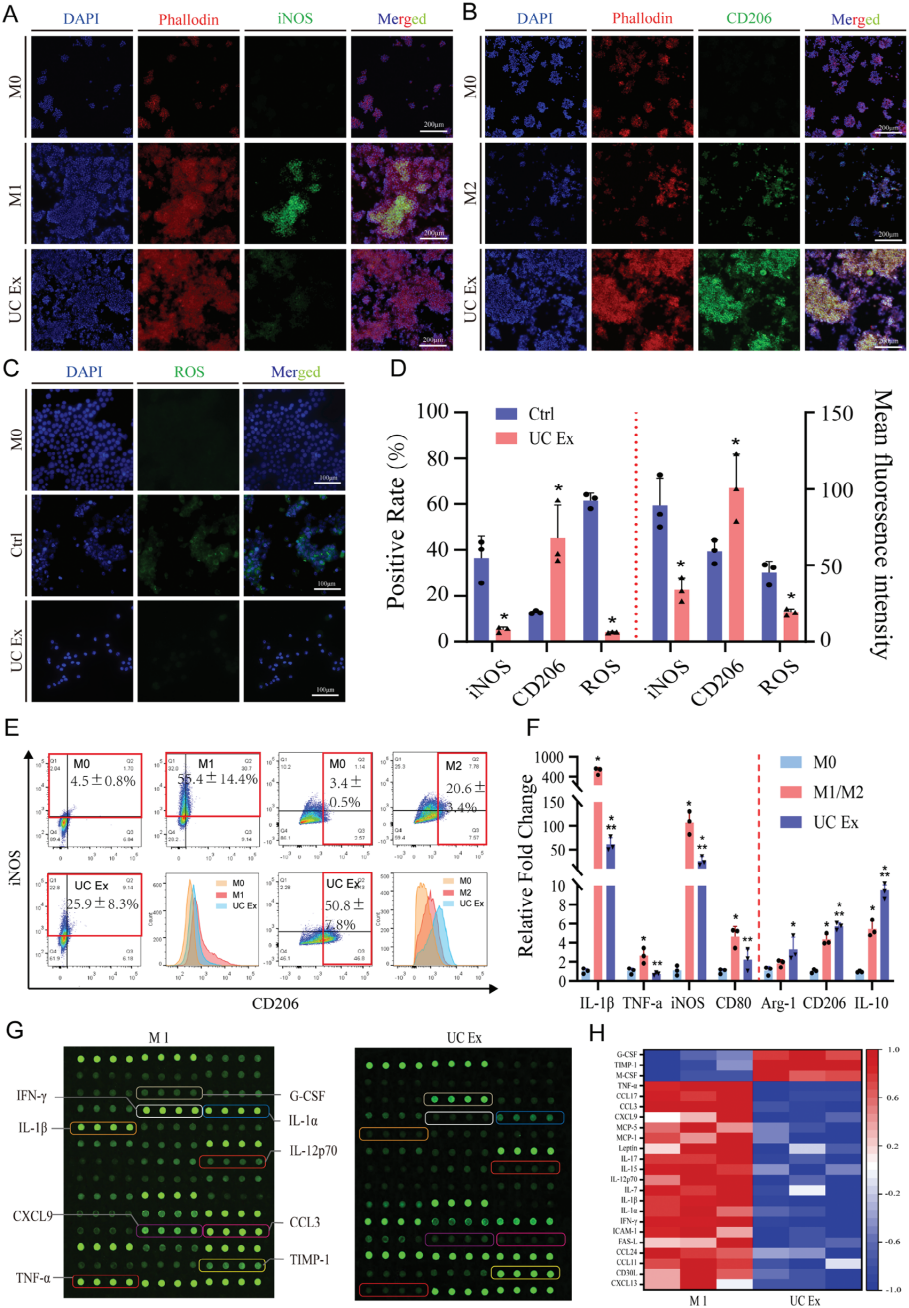
Immunomodulatory effects of UC extracts. A,B) Immunofluorescence staining for iNOS and CD206 in RAW264.7 under different induction conditions(M0: DMEM complete medium; M1/2: DMEM complete medium containing LPS (100 ng mL^−1^) + IFN‐γ (20 ng mL^−1^) or IL‐4 (20 ng mL^−1^)+IL‐13 (20 ng mL^−1^); UC Ex:UC extracts complete medium containing LPS (100 ng mL^−1^) + IFN‐γ (20 ng mL^−1^) or IL‐4 (20 ng mL^−1^)+IL‐13 (20 ng mL^−1^)); C) ROS staining in RAW264.7 under different induction conditions(M0: DMEM complete medium; Ctrl:DMEM complete medium containing 100 ng mL^−1^ LPS + 20 ng mL^−1^ IFN‐γ; UC Ex:UC extracts complete medium containing 100 ng mL^−1^ LPS + 20 ng mL^−1^ IFN‐γ); D) Positive rate and mean fluorescence intensity of Immunofluorescence; E) Flow cytometry of iNOS and CD206 in RAW264.7 under different induction conditions; F) Inflammatory gene expression of RAW264.7 under different induction conditions. G) Inflammatory cytokines chip detection; H) Heatmap of protein secretion in macrophages under different induction conditions. The results are presented as means ± SD. (^*^P < 0.05 compared with control in (D) and compared with M0 in (F), ^**^P < 0.05 compared with M1/M2 in (F).

The immunofluorescence staining results are depicted in **Figure** **4**A,B,D. Following induction of M1 polarization using LPS+IFN‐γ, the control group exhibited a significantly higher proportion of iNOS(+) macrophages (36.4 ± 7.8%) compared to the UC extracts group (5.3 ± 1.0%, P < 0.05). Upon induction with IL‐4+IL‐13, the UC extracts group demonstrated a significantly higher CD206(+) rate compared to the control group (45.2 ± 11.8% vs 12.8 ± 0.5%, P < 0.05), indicating the ability of UC extracts to inhibit M1 polarization and promote M2 polarization of macrophages. Furthermore, the ROS staining (Figure 4C) revealed a significant reduction in ROS expression in macrophages treated with UC extracts compared to the control group (4.0 ± 0.1% vs 61.7 ± 2.6%, P < 0.05), suggesting that UC extracts can effectively attenuate intracellular ROS production in macrophages, thereby potentially contributing to their anti‐inflammatory properties.

The RT‐qPCR results (Figure 4F) also demonstrated that UC extracts can enhance the expression of M2 macrophage‐associated genes while suppressing the expression of M1 macrophage‐associated genes. Further validation of the above findings was performed using flow cytometry, as shown in the Figure 4E. After M1 and M2 induction, the proportion of iNOS + (M1) and CD206 + (M2) macrophages was found to be 55.4 ± 14.4% and 20.6 ± 3.4%, respectively. In the UC extracts group, the proportion of M1 macrophages decreased to 25.9 ± 8.3%, while the proportion of M2 macrophages increased to 50.8 ± 7.8% (comparing to the control group, P < 0.05, respectively).

In addition, we conducted a comparative analysis of the secretion of inflammatory proteins during M1 polarization in the presence or absence of UC extracts (Figure 4G,H). It was noted that during M1 induction, macrophages exhibited a substantial release of pro‐inflammatory cytokines such as IL‐1β and TNF‐α. Conversely, the introduction of UC extracts resulted in a noteworthy decrease in the secretion of pro‐inflammatory proteins. Furthermore, this reduction was accompanied by an augmented secretion of factors including TIMP‐1, M‐CSF, and G‐CSF, which are known to promote tissue regeneration.^[^ ^17^ ^]^

### 2.6 UC Scaffold Promote Chondrogenesis

**Figure 5:**
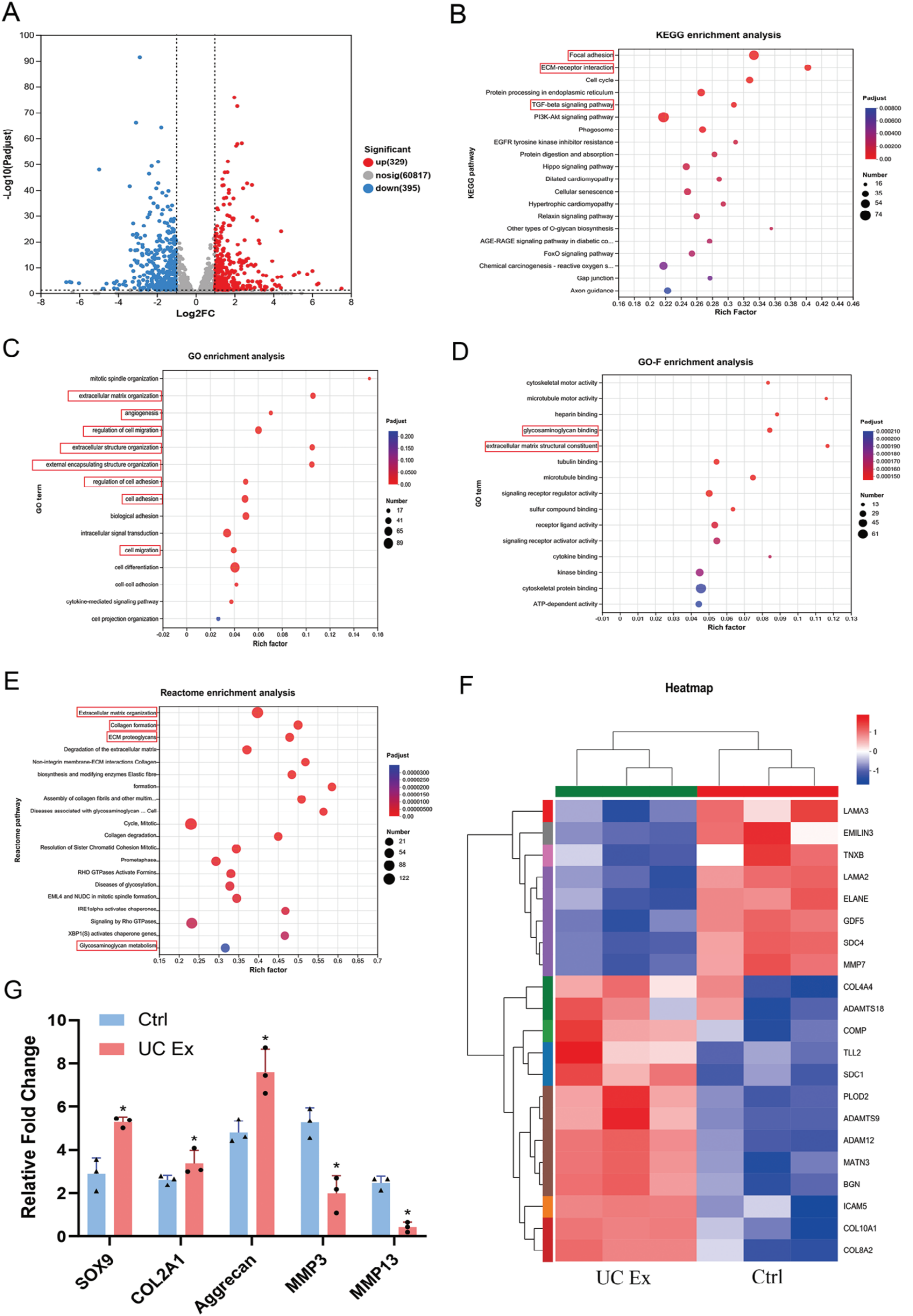
RNA Sequencing of BMSCs cultured in UC extracts chondrogenic medium. A) Volcano plot of differentiated gene expression. B) KEGG enrichment analysis. C) Go enrichment analysis. D) Go‐function enrichment analysis. E) Reactome enrichment analysis. F) Heatmap of differentiated gene expression in Reactome enrichment pathways. G) Chondrogenic gene expression of BMSCs cultured with UC extracts. The results are presented as means ± SD. (^*^P < 0.05 compared with control.).

First, we conducted RNA sequencing analysis on BMSCs cultured in chondrogenic induction medium, as well as BMSCs cultured in chondrogenic induction medium supplemented with UC extracts. The analysis revealed 329 upregulated genes and 395 downregulated genes in the UC extracts group compared to the control group (P < 0.05, |log2| ≥ 1, **Figure** **5**A). Subsequently, we performed enrichment analysis using the KEGG, Reactome, and DO databases to investigate the differentially expressed genes (DEGs). The KEGG analysis (Figure 5B) demonstrated significant differences in the focal adhesion pathway, ECM receptor interaction, and TGF‐β signaling pathway in the UC extracts‐treated group. In the GO analysis, the DEGs in the UC extracts group, when compared to the control group, were primarily associated with biological processes related to cell adhesion, migration, and angiogenesis, as well as molecular functions involving ECM structural components, heparin binding, and cell adhesion (Figure 5C,D). These genes exhibited significant differences between the UC extracts‐treated group and the control group, suggesting that UC extracts can modulate the composition of the ECM and promote cell migration. The Reactome analysis further indicated that UC extracts can regulate processes such as ECM formation, degradation, collagen synthesis, and metabolism. (Figure 5E) Clustering analysis (Figure 5F) of the relevant genes revealed that the UC extracts group showed increased expression of collagen proteins and inhibited synthesis of metalloproteinases, indicating that UC extracts can facilitate chondrogenic differentiation of BMSCs through these mechanisms. Subsequent validation using RT‐qPCR confirmed that UC extracts can upregulate the expression of chondrogenic‐related genes (SOX9, ACAN, COL2) and downregulate the expression of genes related to cartilage metabolism (MMP3, MMP13, P < 0.05, Figure 5G).

**Figure 6:**
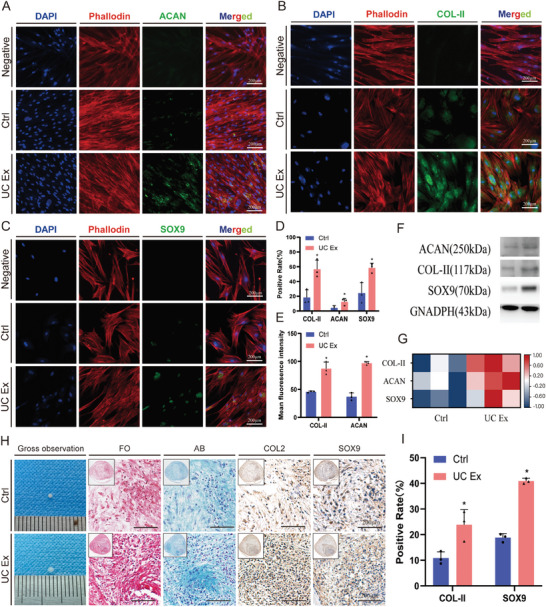
Chondrogenic evaluation of UC extracts. A–C) Immunofluorescence staining for Aggrecan, SOX9 and COL‐II in BMSCs under different induction conditions. D,E) Positive rate and mean fluorescence intensity of Immunofluorescence. F,G) Western‐blot of Aggrecan, SOX9 and COL‐II in BMSCs under different induction conditions. H) Chondrogenic pellet evaluation by staining under different induction conditions. I) Positive rate of immunohistochemistry of pellet staining. The results are presented as means ± SD. (^*^P < 0.05 compared with control.).

We further validated the effect of UC extracts on BMSCs chondrogenic differentiation using immunofluorescence staining (**Figure** **6**A–C) and Western blot (Figure 6E). In the experimental group, there was a significant increase in the proportion of positive cells for ACAN (12.4 ± 3.7%), COL‐II (56.6 ± 2.16%), and SOX9 (58.4 ± 5.2%) compared to the control group (4.2 ± 2.5%, 18.3 ± 8.4%, 24.3 ± 11.5%, P < 0.05 respectively). (Figure 6D) Additionally, the average fluorescence intensity of ACAN and COL‐II was higher in the experimental group compared to the control group (96.8 ± 2.3 vs 36.9 ± 5.5, 87.1 ± 9.6 vs 45.7 ± 1.2, P < 0.05 respectively). (Figure 6E) These findings indicate that ECM enhanced the induction capability of BMSCs toward chondrogenic differentiation, thus providing a favorable in vitro foundation for regenerating the TBI in vivo. The western blot results, as shown in Figure 6F, demonstrated an upregulation of ACAN, COL‐II, and SOX9. The results of relative quantification and normalization analysis, depicted in Figure 6G, further indicated the potential of UC extracts in promoting chondrogenic differentiation of BMSCs.

Chondrogenic pellet is another classic method used to evaluate the chondrogenic differentiation of BMSCs. In this study, we further validate the effect of UC extracts on the chondrogenic differentiation of BMSCs. The macroscopic appearance of the chondrogenic pellets is illustrated in Figure 6H, revealing a larger pellet in the UC extracts group. Furthermore, Alcian Blue and Safranin O staining indicated an increased production of cartilage matrix components in the UC extracts group. Immunohistochemical staining for SOX9 and COL‐II demonstrated a significantly higher positivity rate in the UC extracts group compared to the control group (23.9 ± 4.8% vs 10.9 ± 2.0%, 40.9 ± 0.9% vs 18.8 ± 1.3%, P < 0.05 respectively). (Figure 6H) Additionally, the analysis of GAGs content revealed a significantly higher level in the UC extracts group compared to the control group (Gags/DNA ratio: 9.0 ± 0.5 vs 4.3 ± 0.3, P < 0.05).

### 2.7 Histology and Immunohistochemical Analysis of Canine RC Repair Model

**Figure 7:**
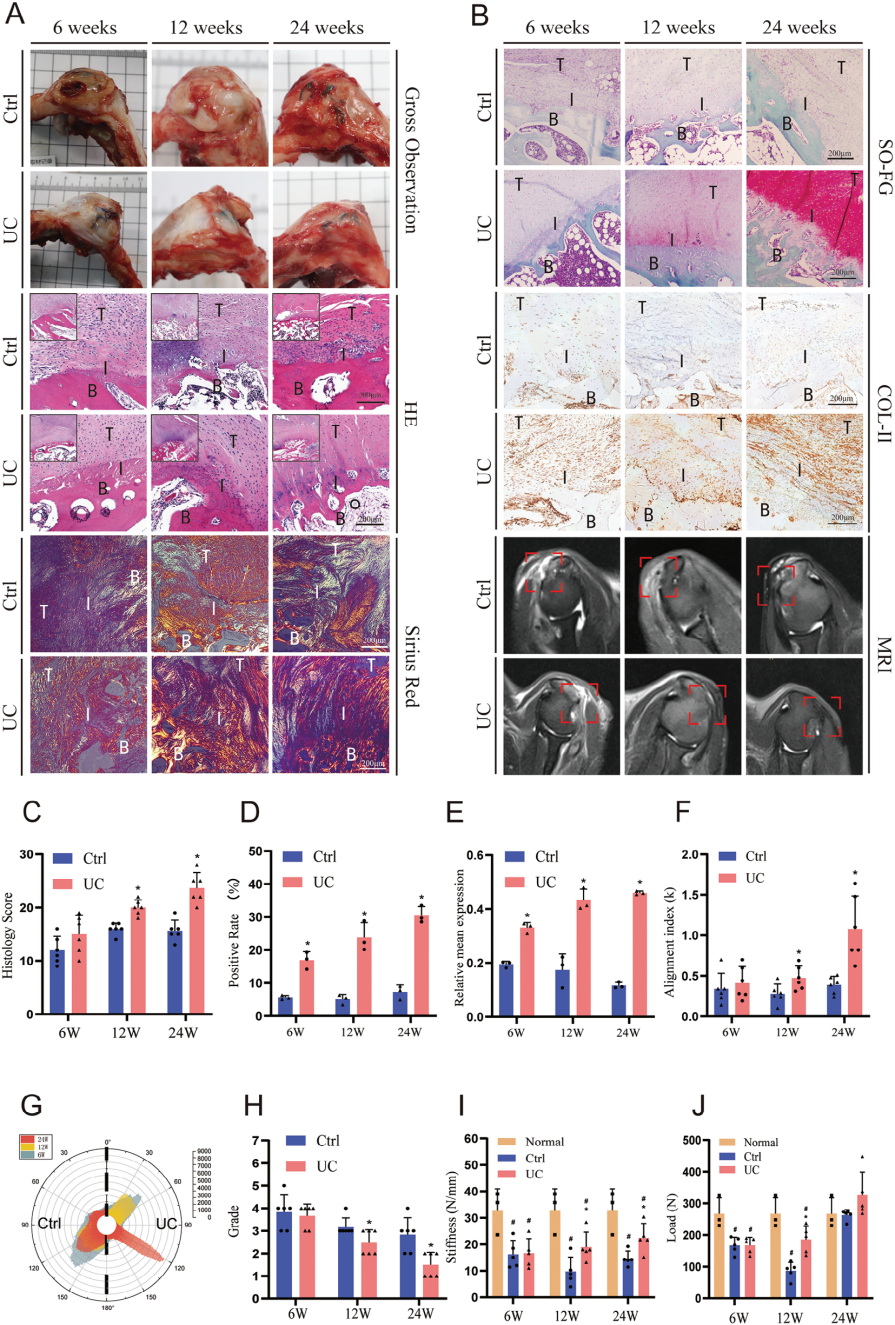
UC Scaffold promotes healing at the tendon‐to‐bone interface. A,B) Representative images of the tendon–bone interface after 6, 12 and 24 weeks. (HE staining, SO staining, Sirius red staining, Col‐II staining, and MRI image. The red rectangle indicates the TBI interface in MRI image. T, tendon; B, bone; I, interface.); C) Histological score of tendon–bone interface; D,E) COL‐II positive rate and relative expression; F,G) Fiber orientation on tendon–bone interface; H) TBI evaluation; I,J) Biomechanical test of the tendon–bone interface. The results are presented as means ± SD. (#P < 0.05 compared with normal; ^*^P < 0.05 compared with control.).

The results of HE staining demonstrated that at 6 weeks, neo‐vascular tissue, fibrous tissue, and inflammatory cells were observed in the control group at TBI. The collagen fibers appeared disorganized with poor alignment. In contrast, the UC group showed increased cellular regeneration, enhanced angiogenesis, presence of fibrous tissue, and less inflammatory cell infiltration. Sirius red staining indicated that the UC group exhibited more formation of type I collagen around the TBI, suggesting a more mature interface compared to the control group (**Figure** **7**A,B).

At 12 weeks, the control group exhibited a reduction in inflammatory cell infiltration. However, collagen fibers remained disrupted, and the TBI showed limited inflammation and localized mild necrosis and neovascularization. In the UC group, there was a decrease in blood vessel distribution and inflammatory cell infiltration. The collagen fibers were more abundant compared to the control group, and their arrangement was more closely aligned and organized. Sirius red staining showed similar results. (Figure 7A,B)

At 24 weeks, the control group showed an increase in the quantity and diameter of collagen fibers, occasionally arranged in a parallel fashion. In contrast, the regenerated tendon structure in the UC group appeared more organized with parallel alignment. Compared to the control group, the collagen fibers in the UC group appeared more compact and parallel, and Sirius red staining indicated a further increase in the presence of type I collagen, suggesting further maturation of the TBI (Figure 7A). The histological scores are presented in Figure 7C. At 6 weeks post‐surgery, there was no significant difference between the control group and the UC group (12.0 ± 2.6 vs 15 ± 3.5, P = 0.128). However, at 12 weeks post‐surgery, the UC group exhibited a significantly higher score than the control group (20.0 ± 1.4 vs 16 ± 1.1, P < 0.01). At 24 weeks post‐surgery, the UC group had a score of 23.7 ± 2.9, significantly higher than the control group's score of 15.7 ± 2.0 (P < 0.01) (Specific details are provided in Table S3, Supporting Information). The immune response triggered by UC scaffold was also evaluated as previously described.^[^ ^18^ ^]^ Limiting tissue reactions between UC group and control group were observed in all periods (6 weeks: P = 0.79; 12 weeks: P = 0.89; 24 weeks: P = 0.20 Tables S4 and S5, Supporting Information).

Immunohistochemical staining for COL‐II and Safranin O‐Fast Green staining were performed to assess the cartilage regeneration of TBI. (Figure 7A,B) The UC group consistently demonstrated significantly elevated levels of COL2 expression compared to the control group at all times. (Figure 7D,E) Collagen alignment evaluation was presented in Figure 7G,H. As depicted, fibers were more organized in UC group after 12 and 24 weeks (P < 0.05). Furthermore, Safranin O‐Fast Green staining revealed a higher abundance of cartilaginous matrix components in the UC group. These findings suggest that the implantation of the UC patch promotes the development of interface cartilaginous components.

### 2.8 MRI Analysis

As depicted in Figure 7B, at 6 weeks postoperatively, the control group exhibited a higher TBI signal, indicating more edema and inflammatory response, along with continuity between the tendon and bone. Similar results were observed in the UC group, and the MRI grade between the two groups did not show statistical differences (3.8 ± 0.7 vs 3.7 ± 0.5, P = 0.71, Figure 7H). At 12 weeks postoperatively, the interface signal remained high and disorganized in the control group, whereas in the UC group, the TBI signal decreased, with a thinner tendon compared to normal, and continuity between the tendon and bone. The UC group demonstrated superior MRI grade compared to the control group (2.5 ± 0.5 vs 3.2 ± 0.4, P = 0.04, Figure 7H). By 24 weeks postoperatively, the TBI signal further decreased in the control group, with increased tendon thickness, but still with localized high signal areas. In contrast, the UC group exhibited a further reduction in the tendon–bone interface signal, increased thickness, resembling a normal tendon–bone interface. The MRI grades in the UC group were superior to the control group (1.5 ± 0.5 vs 2.8 ± 0.7, P = 01, Figure 7H).

### 2.9 Biomechanical Analysis

Biomechanical evaluations were conducted at 6, 12, and 24 weeks postoperatively, as shown in Figure 7I,J. The normal canine RC demonstrated a failure load of ≈267.4 ± 50 N and a stiffness of ≈32.8 ± 8.1 N mm^−1^. At 6 weeks postoperatively, there were no statistically significant differences in failure load and stiffness between the control and UC groups (167.3 ± 25.9 vs 167.6 ± 25.2 N, P = 0.99; 16.2 ± 5.2 vs 16.64 ± 5.5 N mm^−1^, P = 0.92), but both were lower than the normal group (P < 0.05). At 12 weeks postoperatively, the mechanical performance of the control group decreased compared to the previous assessment, while the UC group exhibited improved mechanical strength and outperformed the control group (87.4 ± 26.5 vs 186.0 ± 42.9 N, P < 0.01; 10.5 ± 7.0 vs 18.5 ± 6.3 N mm^−1^, P = 0.1). At 24 weeks postoperatively, there were no significant differences in failure load between the UC group (326.0 ± 73.1 N) and the normal group (P = 0.16) or the control group (263.2 ± 16.5 N, P = 0.08). The stiffness of the UC group (22.4 ± 5.5 N mm^−1^) was superior to that of the control group (15.4 ± 4.3 N mm^−1^, P = 0.08), but still lower than that of the normal RC tissue.

### 2.10 Early Outcome of a Clinical Trial

**Figure 8:**
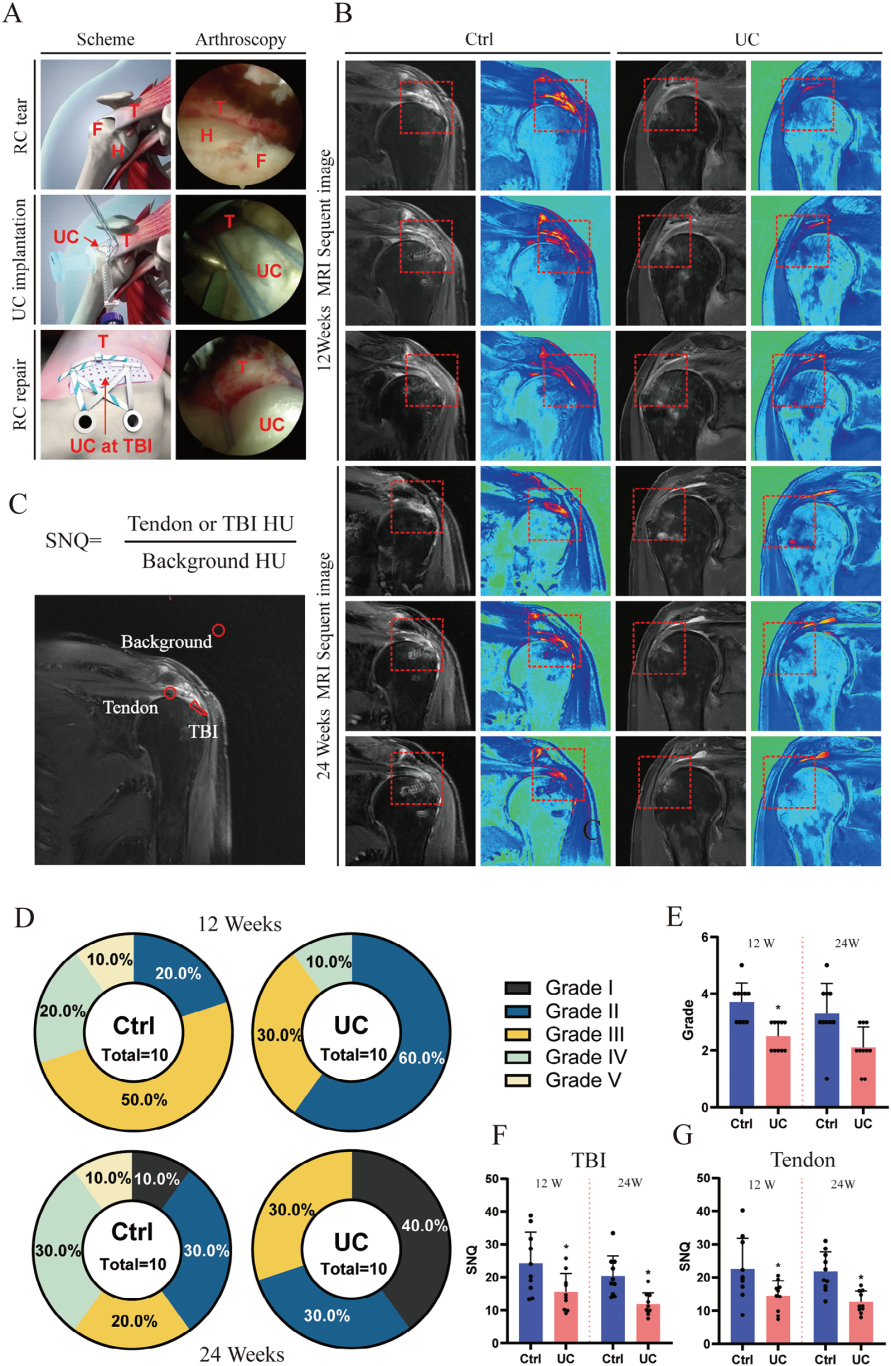
Early outcome of UC scaffold implantation in patients. A) Procedure of UC scaffold implantation under arthroscopy surgery. B) Representative sequent MRI images after 12 and 24 weeks implantation; C) Signal to noise measurement of tendon–bone interface and tendon; D) TBI evaluation by Sugaya grade; E) MRI grade of TBI; F/G. TBI and tendon SNQ evaluation. The results are presented as means ± SD. (^*^P < 0.05 compared with control.).

The surgical procedure was conducted as shown in **Figure** **8**A. An arthroscopic examination was performed to determine the size of the RC injury, and patients meeting the inclusion criteria were randomly assigned to either the control group or the experimental group. The control group underwent double‐row repair, while the experimental group received an additional patch implantation at the site of the TBI before fixation. For this study, the first 20 patients who met the clinical trial inclusion criteria were included, with 10 patients in the control group and 10 patients in the experimental group. As depicted in Figure 8B, the control group exhibited higher signal intensity at the TBI site 12 and 24 weeks after direct repair, indicating a stronger inflammatory reaction, whereas the UC implantation group showed lower signal intensity. At 12 weeks post‐surgery, the Sugaya classification in the control group (as shown in Figure 8D) consisted of 2 cases in Grade II, 5 cases in Grade III, and 2 and 1 cases each in Grade IV and Grade V, whereas the UC group had 6 cases in Grade II, 2 cases in Grade III, and 2 cases in Grade IV. Further healing process was observed after 24 weeks post‐surgery, 40% of the patients reached Grade I in UC group indicating the best healing effect. Grades I–III were categorized as the healing group, and Grades IV–V were classified as the tear group for comparison, with no significant difference observed between the two groups' effects (12 weeks: P = 0.58; 24 weeks: P = 0.09). Although MRI grade did not show a significant difference at 24 weeks (Figure 8E and P = 0.06), early healing was confirmed at 12 weeks (P < 0.01). The SNQ (signal‐to‐noise ratio) measurement results, as shown in Figure 8F,G, indicated significantly lower SNQ values in the TBI and T areas of the UC implantation group compared to the control group (12 weeks: P = 0.02, P = 03; 24 weeks: P < 0.01, P < 0.01), demonstrating that UC implantation effectively reduces post‐operative inflammatory reactions during the repair process.

## 3 Discussion

In this study, we developed a scaffold derived from human UC. We validated its capability to enhance RC repair through in vitro experiments and large animal model. Besides, short‐term outcome of the clinical trial was also reported, providing effective evidence of the advantage of UC scaffold implantation. This research provides essential scientific groundwork for the translational application of UC scaffolds in clinical settings. Initially, we conducted mass spectrometry analysis to characterize the scaffold's protein composition. In vitro cell evaluations were performed to assess its biocompatibility, immune modulation, and chondrogenic differentiation potential. It was observed that the scaffold might promote BMSC chondrogenic differentiation by influencing ECM metabolism, leading to cartilage formation. Subsequently, we established a large animal model of RC tear, followed by repair surgery. Histological, biomechanical, and MRI evaluations confirmed that scaffold implantation led to the formation of a more mature TBI structure. Ultimately, the short‐term outcomes of the clinical trials demonstrated that UC scaffold implantation could facilitate the development of TBI healing.

One of the key issues determining the success of RC repair lies in the regeneration of the TBI, which is critical for achieving a favorable outcome.^[^ ^19^ ^]^ The normal TBI is a complex structure composed of four transitional zones: “bone‐calcified fibrocartilage‐non‐calcified fibrocartilage‐tendon.” This unique structure allows for the dispersion of stress throughout the entire interface, thereby preventing tendon rupture resulting from localized stress concentration.^[^ ^20^ ^]^ However, after RC repair, the regenerated structure at the TBI typically consists of fibrous tissue, blood vessels, and scar tissue, which possess lower mechanical strength compared to the normal interface. Consequently, the repaired interface is prone to re‐tearing.^[^ ^21^ ^]^ Promoting the regeneration of the TBI is a crucial challenge that we urgently need to address.

The current study suggests that UC harbors a wealth of bioactive constituents, including collagen, TGF‐β, periostin, fibronectin, and GAGs. Functioning as a 3D porous biomaterial, it facilitates nutrient transfer and transport, with remarkable biocompatibility. Additionally, it modulates the behavior of BMSCs by promoting adhesion, migration, proliferation, and recruitment. Consequently, the UC holds great promise as a tissue engineering scaffold for tissue repair. It has found widespread applications in various tissue engineering endeavors, such as wound healing,^[^ ^11^ ^]^ cartilage repair,^[^ ^13^ ^]^ tracheal repair,^[^ ^22^ ^]^ and RC repair.^[^ ^14^ ^]^

Decellularization techniques have been extensively employed in recent decades to mitigate the immunogenicity of biomaterials. However, the decellularization process inevitably results in the loss of certain components from the material. Dubus et al. successfully developed a decellularized UC matrix that effectively preserved ECM components, demonstrating insignificant alterations in collagen and GAGs compared to the native matrix.^[^ ^11^ ^]^ Conversely, another study employing proteomic analysis identified some loss of bone and cartilage‐related factors after decellularization.^[^ ^23^ ^]^ In this study, we adopted a minimally processed approach to safeguard the structure and composition of the UC tissue. Furthermore, the UC is recognized as an immunoprivileged tissue with low‐immunogenicity. Non‐decellularized materials or cell‐based products derived from the UC have been clinically applied without substantial immune rejection, including wound healing^[^ ^24^ ^]^ and tendon repair.^[^ ^25^ ^]^ In our research, histological analysis further confirmed the absence of significant immune rejection, underscoring its low immunogenicity characteristics.

Increasing evidence suggests that the immune system plays a crucial regulatory role in tissue regeneration across various tissues.^[^ ^26^ ^]^ Both sides of the torn RC remnants undergo infiltration by inflammatory cells.^[^ ^27^ ^]^ Inflammatory factors secreted by these cells, such as IL‐1β and TNF‐α, may activate the NF‐κB pathway, which potentially impacts TBI healing by promoting osteoclast differentiation, increasing matrix metalloproteinase synthesis, suppressing SOX9 expression, and inhibiting collagen production.^[^ ^28^ ^]^ Consequently, several studies have explored the use of various anti‐inflammatory agents to promote TBI healing.^[^ ^28^, ^29^ ^]^ Wharton's jelly (WJ), a major component and structure within the UC, is also a biobank of MSCs. Multiple studies have highlighted the potent immunomodulatory potential of UC‐derived MSCs, primarily mediated through the secretion of bioactive factors.^[^ ^11^ ^]^ WJ is rich in these secreted factors, as demonstrated by Gupta et al., who utilized UC‐derived functionalized silk vascular grafts for vascular reconstruction. Their studies revealed that the UC extracts upregulated M2 macrophage‐related genes. M2 macrophages, in turn, induce tissue remodeling by regulating fibrosis and ECM component deposition.^[^ ^11^, ^30^ ^]^ Similarly, another study indicated that decellularized WJ exhibited immunomodulatory effects on macrophages.^[^ ^11^ ^]^ In our study, we induced RAW267.4 cells with UC extract and observed inhibition of M1 macrophage polarization along with promotion of M2 macrophage differentiation. Co‐culture experiments further revealed a reduction in pro‐inflammatory cytokine secretion, indicating excellent immunomodulatory capacity. Moreover, we observed an increase in TIMP‐1 levels, effectively inhibiting MMP production, reducing extracellular matrix degradation, and promoting collagen deposition.^[^ ^31^ ^]^ This may be one of the primary mechanisms facilitating TBI repair.

Angiogenesis is another crucial factor required for tendon–bone healing.^[^ ^32^ ^]^ Although the normal TBI is considered avascular, sufficient blood supply after injury is vital for the delivery of nutrients, minerals, and oxygen necessary for bone synthesis and mineralization, as well as maturation of the tendon matrix. In patients with RC tears, the blood supply to the tendon insertion is disrupted, which is considered one of the most significant factors contributing to incomplete healing and re‐tear.^[^ ^33^ ^]^ In our study, the results suggest that UC extracts promote HUVECs tube formation and enhances the expression of angiogenesis‐related genes.

WJ has emerged as a highly regarded natural scaffold for cartilage tissue engineering due to its abundant supply of cartilage‐specific ECM components.^[^ ^13^, ^22^ ^]^ Moreover, the presence of MSCs within WJ signifies its potential to create an optimal microenvironment conducive to the survival and functionality of stem cells. In line with previous research, our current findings demonstrate the substantial presence of GAGs and collagen in WJ, both of which are major constituents of cartilage tissue. Additionally, WJ contains various growth factors, including IGF‐I and TGF‐β, which actively promote cartilage formation. Prior studies have successfully utilized UC for cartilage regeneration.^[^ ^13^, ^22^ ^]^ In our investigation, we observed that UC extracts upregulated the expression of genes and proteins associated with cartilage development (SOX9, COL‐II, ACAN). RNA sequencing analysis revealed significant enrichment changes in pathways related to collagen synthesis, ECM production, cell adhesion, migration, and other pertinent processes subsequent to UC extracts. Heatmaps exhibited elevated expression levels of cartilage‐related factors, such as cartilage oligomeric matrix protein (COMP) and collagen, indicating the potential mechanisms. The facilitation of cartilage formation was further substantiated through cell immunofluorescence and cartilage pellet analysis.

In recent years, there has been a growing body of research focused on the utilization of UC for RC repair. Yuan et al. developed a decellularized UC scaffold for RC repair. Their findings suggested that the decellularized UC patch exhibited good biocompatibility with the potential to promote RC healing.^[^ ^14^ ^]^ Furthermore, kartogenin was further loaded on the scaffold to enhance TBI regeneration.^[^ ^23^ ^]^ However, despite the promising outcomes obtained in small animal models, further research in large animal models is necessary before clinical application. Large animal models are more similar to clinical scenarios in having poorer self‐healing capabilities for tissue injuries compared to small animal models.^[^ ^34^ ^]^ The canine model is one of the mature and widely used large animal models for tendon–bone regeneration.^[^ ^7^, ^10^, ^35^ ^]^ In this study, we established a canine model of RC repair and conducted long‐term observations (24 weeks) of the scaffold implanted at the TBI. The histological results were consistent with the conclusions obtained from in vitro experiments. The implantation of the UC patch promoted the regeneration of the cartilage layer at the TBI, thereby providing stronger mechanical strength.

Currently, there are only two commercially available scaffolds for TBI repair. These products are primarily composed of decalcified bone‐derived materials or biodegradable synthetic materials. As of now, no clinical studies have been reported for the former. Seetharam et al., on the other hand, presents a retrospective investigation involving the implantation of a nanofiber scaffold for the treatment of small to medium‐sized RC tears. By introducing PLCL (poly(L‐lactide‐co‐ε‐caprolactone)) at the TBI to facilitate healing, the study reports a surgical failure rate of 9%, which is primarily observed in patients with larger tears.^[^ ^36^ ^]^ However, this synthetic material lacks bioactive components. In this study, we present, for the first time, clinical imaging data of a bioactive scaffold implanted at the TBI of patients with large RC tears. The results indicate a significant reduction in signal intensity at TBI 12 and 24 weeks after implantation. This reduction suggests a lower inflammatory response in the surrounding tissues and a more mature interface structure.

Our current study has certain limitations: First, the anatomical structure and movement patterns of the shoulder in canine model differ from those in humans, and the healing environment of the RC tear cannot be fully replicated. Additionally, the clinical trials yielded only a limited number of short‐term research outcomes. Further investigation is required to more effectively substantiate the observed effects. Due to a lack of suitable antibodies, the histological analysis methods were limited, and the validation of inflammation and cartilage‐related markers could not be confirmed. Second, the animal model established represents an acute injury model, which cannot simulate the changes observed in clinical with chronic RC tears, tendon degeneration, or fatty infiltration. However, establishing a chronic RC tear model in animal model may lead to variability due to surgical differences and individual variations. Furthermore, we performed bilateral surgical procedures in the experiments, which may have partially influenced the animal's activity. The impact of this on the experimental results remains unknown, but previous studies have also employed bilateral surgery treatment, suggesting limited effects.^[^ ^10^ ^]^ Umbilical cord derived scaffolds contain several bioactive components, the specific proportion of each factor was not measured in this study. Further exploration is needed to investigate the mechanisms underlying the promotion of TBI healing by the UC scaffold.

## 4 Conclusion

In conclusion, this study demonstrates that the UC scaffold contains various bioactive components, exhibits good biocompatibility, and supports cell adhesion and proliferation. Furthermore, the scaffold has anti‐inflammatory effects by inhibiting pro‐inflammatory macrophage differentiation and inflammatory factor secretion while promoting anti‐inflammatory macrophage differentiation and facilitating angiogenesis. Mechanistic analysis reveals that the UC scaffold promotes the chondrogenic differentiation of BMSCs by influencing ECM synthesis, deposition, and metabolism, thereby facilitating TBI regeneration. Using the canine RC repair model, we have validated the ability of the UC scaffold to reconstruct the complex structure of the TBI, providing a solid and effective basis for clinical applications. Lastly, the short‐term results of the clinical trial indicate that UC scaffold implantation can facilitate early‐stage maturation of the TBI.

## 5 Experimental Section

### Scaffold Preparation

The human UC scaffold was provided by a qualified commercial company (Dongzhiyixue Ltd, Shanghai). Under low‐temperature conditions, the UC tissue was rinsed with PBS to remove blood and tissue fluid. The UC was longitudinally cut, and physical methods such as scissors and round drills were employed to remove vascular and other tissues. The remaining tissue was trimmed to a size of 2 × 6 cm, placed in a rectangular carrier, and subjected to a freezing process at −20 °C for 4 h, followed by freezing at −80 °C for 20 h. After freeze‐drying for 24 h, the UC scaffolds were prepared and subjected to irradiation sterilization.

### Physicochemical Characterization of UC Scaffold—Hydrophilicity Evaluation and Mechanical Analysis

The hydrophilicity of UC scaffold was measured. Deionized water was manually dropped on the UC scaffold. The process was recorded and analyzed by Goniometer (OCA40, Data‐physics Instruments, Filderstadt, Germany). The UC scaffold was shaped into 30 mm × 10 mm and stretched at 5 mm min^−1^ by material testing machine (5569R, Instron.). Failure load and strain at break were analyzed.

### Physicochemical Characterization of UC Scaffold—Protein Analysis

The proteomic component of UC scaffold was analyzed by mass spectrometry. Briefly, the peptide solution was prepared by a filter‐aided sample preparation (FASP)protocol. The UC scaffold was lysed using a solution containing 4% SDS, 100 mm Tris‐HCl, 1 mm DTT, pH 7.6. The protein concentration was measured using the BCA assay, and 200 µg of protein was used for subsequent experiments. After protein sample processing using the FASP method, LC‐MS/MS analysis and protein identification were performed using the Q Exactive mass spectrometer (Thermo Scientific) coupled with Easy nLC (Proxeon Biosystems, Thermo Fisher Scientific) for 60 min. The protein identification was searched using the MaxQuant 1.5.3.17 software. Two of the top 30 coverage protein detected in mass spectrometry were evaluated by Elisa assay. Briefly, UC scaffold was immersed in PBS at 37 °C and 70 rpm with supernatants collected at different time points (1, 7, 14, 21, and 28 days), followed by procedures described in manual instructions of the Elisa kits (Solarbio, SEKH‐0103; Cusabio, CSB‐E16375h).

### Physicochemical Characterization of UC Scaffold—GAGs and Collagen Content Analysis

The GAG content of the UC scaffold was measured using Blyscan Glycosaminoglycan kit (Biocolor Ltd, Carrickfergus, UK), while the collagen content was determined using a hydroxyproline assay kit (Nanjing jiancheng Bio Int. Nanjing, China), following the manufacturer's instruction. For GAG analysis, UC scaffold was lysed by Papain extraction reagent for overnight at 60  °C before measurement. For Hydroxyproline assay, the UC scaffold was lysed by 6 m HCL for 5 h at 100 °C. The absorbance in standards and samples was measured at 656 and 550 nm with microplate reader.

### Physicochemical Characterization of UC Scaffold—Scanning Electron Microscopy (SEM), HE, and Masson Staining

The UC scaffold was fixed with glutaraldehyde for 24 h. After gradient dehydration using ethanol, it was subjected to critical point dryer and gold coating for SEM examination (JEOL, Japan). For histology staining, the scaffold was immersed in 4% paraformaldehyde for 2 days, followed by embedding in paraffin and cutting into 5 µm thick sections. These sections were then subjected to HE and Masson staining.

### Biocompatibility—Preparation of UC Extracts

UC scaffold were sterilized by UV irradiation. The sterilized UC scaffold was immersed in Dulbecco's modified Eagle's medium (DMEM) at 37 °C for 72 h. Then, the conditional medium was centrifuged at 1000 rpm for 5 min. The supernatant was collected and sterilized with a 0.22 mm filter membrane (Millipore) to prepare the UC extracts.

### Biocompatibility—Cell Proliferation and Viability

Human bone marrow‐derived mesenchymal stem cells (BMSCs) were obtained from OriCell and seeded at a density of 1000 cells per well in a 96‐well plate. The culture medium was replaced with either DMEM or UC extracts, with 10% fetal bovine serum (FBS), and cultured for 1, 3, or 5 days. Cell proliferation was assessed using a cck‐8 assay kit (Beyotime, Shanghai, China). In brief, cck‐8 working solution was added to each well and incubated at 37 °C for 1.5 h. The supernatant was then collected, and the absorbance at 450 nm was measured by UV spectrophotometer. For cell viability, BMSCs were seeded (10 000 cells per well) in a 24‐well plate. The cells were cultured in DMEM or UC extracts with 10% FBS at 37 °C and 5% CO_2_, for 1, 3, and 5 days. Cell viability was assessed using a live/dead staining kit (Beyotime, Shanghai, China).

### Biocompatibility—Cell Morphology on UC Scaffold

The UC scaffold was cut into 1 cm diameter circles and immersed in DMEM medium overnight. Then, 5000 cells per well were seeded onto the scaffold and cultured for 5 days at 37 °C and 5% CO2. For SEM, it was carefully washed three times with PBS, followed by the same process as previously described. For cell cytoskeleton observation, the samples were fixed with 4% paraformaldehyde, treated with 0.2% Triton X‐100 for 10 min, incubated with 5% BSA for 30 min, sequentially stained with rhodamine phalloidin and DAPI for 30 min each, and then observed using a confocal microscope (Leica, Germany).

### Biocompatibility—Cell Migration Analysis

Cell migration was evaluated using the Transwell and scratch tests. BMSCs were seeded at a density of 10 000 cells per well in upper chambers. After 8 h, the medium was replaced with serum‐free DMEM for overnight. The upper chamber was maintained with serum‐free medium, while the lower chamber received DMEM or UC extracts with 10% FBS. After 24 h, residual cells on the upper surface of the chambers were removed using a cotton swab, and crystal violet staining was performed for subsequent analysis.

In the scratch test, BMSC cells were seeded in a 6‐well plate and allowed to reach ≈90% confluence. The medium was then replaced with serum‐free DMEM for 12 h. A scratch was made with a 200 µL pipette tip, and serum‐free medium or UC extracts were added. The area of the scratch was observed at 0, 24, and 48 h to assess cell migration.

### Angiogenesis Analysis

To assess the angiogenic capacity, the tube formation assay and RT‐qPCR were performed. Before cell seeding, 10 µL of Matrigel was added to each well of 15‐well µ‐Slide (Ibidi, Germany) kept at 37 °C for 30 min. 5000 human umbilical vein endothelial cells (HUVECs) were grown in each well with DMEM or UC extracts complete medium. After 8 h of incubation, the samples were observed under a light microscope.

For RT‐qPCR, HUVECs were cultured in 6‐well plates and allowed to reach confluence. Then, DMEM complete medium and UC Extracts‐conditioned medium were added and the cells were cultured for 24 h. Subsequently, RT‐qPCR was performed to measure the expression of angiogenesis‐related genes.

### Anti‐Inflammation Analysis—Raw264.7 Polarization

RAW 264.7 cells were seeded in a 6‐well plate. For M1 polarization induction, DMEM complete medium and UC Extracts medium containing LPS (100 ng mL^−1^) + IFN‐γ (20 ng mL^−1^) were separately added and cultured for 48 h. For M2 polarization induction, DMEM complete medium and UC Extracts medium containing IL‐4 (20 ng mL^−1^)/IL‐13 (20 ng mL^−1^) were separately added and cultured for 48 h.

### IAnti‐Inflammation Analysis—Immunofluorescence Staining

Immunofluorescence staining was performed to evaluate the effect of UC Extracts on macrophage polarization. RAW 264.7 cells were fixed with 4% paraformaldehyde, permeabilized with 0.2% Triton‐X for 10 min, and blocked with 5% BSA, followed by primary antibodies incubation (Dilution: 1:200, iNOS: ab178945; CD206: ab64693, Abcam) overnight at 4 °C and Alexa Fluor 488‐conjugated secondary antibody (Abcam, ab150077, UK) for 1 h. Subsequently, cells were stained with Rhodamine Red‐labeled phalloidin and DAPI for 30 min each, and observed under a fluorescence microscope.

### Anti‐Inflammation Analysis—Reactive Oxygen Species (ROS) Assay

The production of ROS in RAW264.7 was assessed using an ROS assay kit (Beyotime, Shanghai, China). After removing the M1‐inducing medium, the working solution was added and incubated at 37 °C for 30 min. The cells were then washed with PBS and observed and analyzed under a fluorescence microscope.

### Anti‐Inflammation Analysis—Inflammatory‐Related Gene Expression and Flow Cytometry

The expression of inflammatory‐related genes (M1: IL‐1β, TNF‐α, iNOS, CD80; M2: Arg‐1, CD206, IL‐10) were analyzed using rt‐qPCR following standard protocol. The primer sequences are presented in Table S1 (Supporting Information). In flow cytometry, the polarized RAW264.7 cells were washed three times with PBS, followed by 0.5% Triton X‐100 for 10 min and 3% BSA for 15 min. The cells were incubated with flow cytometry antibodies for iNOS (BioLegend) and CD206 (BioLegend) at a dilution of 1:500 for 30 min. Subsequently, flow cytometry analysis was conducted using a flow cytometer, and further analysis was performed using FlowJo software.

### Anti‐Inflammation Analysis—Inflammatory Factors Secretion

The supernatant from M1‐induced cell was collected, and quantitative detection of inflammatory factors was performed using the Mouse Inflammation Array Q kit (RayBiotech, QAM‐INF‐1) following the manual instructions.

### Chondrogenesis Assay—RNA‐Sequence and RT‐qPCR

BMSCs were induced for chondrogenic differentiation for 7 days with DMEM and UC extracts containing 100 nm dexamethasone, 50 µg mL^−1^ ascorbic acid 2‐phosphate, 1 mm sodium pyruvate, 40 µg mL^−1^ proline, 10 ng mL^−1^ recombinant human transforming growth factor‐β3 (TGF‐β3), and 1:100 diluted ITS Premix. The total RNA was extracted by TRIzol reagent and sent to Majorbio Cloud for further analysis.^[^ ^37^ ^]^ The expression of chondrogenic genes (SOX9, COL2A1, Aggrecan, MMP3, MMP13) was analyzed using RT‐qPCR following standard protocol. The primer sequences are presented in Table S1 (Supporting Information).

### Chondrogenesis Assay—Chondrogenic Evaluation

For Immunofluorescence and western blot analysis, BMSCs were seeded in 24‐well plates and 6‐well plates respectively. Then, the culture medium was replaced with chondrogenic induction medium. The medium was refreshed every three days, and the cells were cultured at 37 °C with 5% CO2 for 14 days. Afterward, the cells were fixed and subjected to immunofluorescent staining and western blot for Aggrecan (DF7561, affinity), SOX9(A19710, Abclonal), and COL‐II (A1560, Abclonal).

### Chondrogenesis Assay—Pellets Analysis

Centrifuge 5 × 10^5^ BMSCs at 200 g for 5 min in a 15 mL centrifuge tube and incubate at 37 °C with 5% CO2 for two days to form cell pellets. Then, replace the culture medium with either chondrogenic induction medium or UC extracts chondrogenic induction medium and refresh the medium every three days until 21 days. GAGs, histology, and immunohistochemistry (IHC) analysis were performed. In GAGs analysis, the pellets were lysis and measured as previously described. For histology and IHC analysis, the pellets were immersed in O.C.T. compound overnight before freezing, and subsequently, tissue sections were prepared for Safranin O staining, Alcian Blue staining, and IHC (Coll‐II, Aggrecan, and SOX9).

### Canine Rotator Cuff Repair Model—Study Design

This study was conducted in accordance with the ethical standards and guidelines set by the Animal Management and Use Committee of Suzhou Zhenhu Medical Technology Co., Ltd. (Approval No. 110‐2020‐03), which was funded by Dongzhiyixue Ltd, which was not involved in the data collection, data analysis, and preparation and editing of the manuscript. A total of 36 beagle dogs were randomly divided into three groups (6, 12, and 24 weeks) receiving bilateral RC tear and repair surgery. RC repair and UC scaffold implantation were performed on left shoulder, while the other side only received RC repair. Histology (HE, Masson, Sirius red, Safranin O‐Fast Green, and immunohistochemistry staining), radiology, and biomechanical evaluation were conducted. Fiber orientation at TBI was evaluated as previously described through Sirius red staining.^[^ ^38^ ^]^

### Canine Rotator Cuff Repair Model—Surgery Procedure

Longitudinal incision was created on the anterolateral side of the shoulder. Subcutaneous connective tissue and the deltoid muscle were bluntly separated to expose the infraspinatus tendon (IST) at the humeral greater tuberosity. The IST was completely detached at its footprint, followed by removing the tendon remnants and cartilage layer on bone side. Two 2 mm diameter bone tunnels were created by k‐wire, on both sides of the insertion point. Non‐absorbable sutures were passed through the bone tunnels and tendon remnants to reattach the torn tendon. For the experimental group, the UC scaffolds were placed between the tendon and bone before knotting. After irrigation with physiological saline, the separated deltoid muscle and skin incision were sutured layer by layer.

### Canine Rotator Cuff Repair Model—MRI Analysis

After sacrifice, MRI was performed on each shoulder using 3.0 Tesla, GE signa HDX, and a shoulder coil. T1 with fat saturation(FS; thickness 3 mm; matrix, 192 × 256, TR 517 ms, TE 15 ms), T2 FS(thickness 3 mm; matrix, 192 × 256, TR 2500 ms, TE 94 ms) and proton density FS sequences (thickness 3 mm; matrix, 192 × 256, TR 2102 ms, TE 18 ms) were applied. TBI evaluation was conducted by 2 examiners blinded to the group, as the following table (**Table** **1**).^[^ ^39^ ^]^

**Table 1:** TBI evaluation (MRI grade).

Grade	Description
I	Signal similar to tendon
II	Signal greater than tendon but less than muscle
III	Signal similar to muscle
IV	Signal greater than muscle but less than joint fluid
V	Signal similar to joint fluid

### Canine Rotator Cuff Repair Model—Biomechanical Test

IST was braided with a polyester cloth for the fixation on the clamp, and the humerus was clamped on the other side, which was confirmed with sufficient strength for mechanical testing for the canine RC in our pilot study. Ensuring that the tendon was perpendicular to the longitudinal axis of the humerus. The samples were subjected to a tensile test by applying a preload of 5 N, followed by a fixed displacement rate of 5 mm min^−1^ until tendon failure. Load and displacement data were collected to determine the maximum failure load and stiffness.

### Early Evaluation of UC Scaffold Implantation in Patients

A multi‐center prospective open‐label clinical trial was conducted (ChiCTR2100050630). The study design and the informed consent document were approved by the institutional review board of Shanghai Sixth People's Hospital. Patients with RC tear were recruited in accordance with the following inclusion criteria: 1) age over 18 years; 2) diagnosed with large tear (RC tear over 3 cm). Exclusion criteria included: 1) patients with Alzheimer's disease or dementia who are too delirious to make subjective assessments; 2) participants in another clinical trial within 6 months; 3) pregnancy, lactating woman; 4) shoulder osteoarthritis; 5) humeral fracture; 6) shoulder instability history; 7) severe systemic or local infection. The patients were randomized into control group or UC implantation group, and underwent double‐row arthroscopic RC repair while only the UC group was implanted with UC scaffold at TBI. The folded UC scaffold was delivered and expanded on the RC footprint by a customized device before lateral anchor fixation. Immobilization began immediately until 6 weeks after the surgery, following by passive and active rehabilitation with standardized protocol of each center. Early outcome was evaluated with Sugaya grade (**Table** **2**), MRI grade and the signal/noise quotient (SNQ) of the region of interest (ROI) through MRI by 2 independent observers (blinded to the treatment group) after 12 and 24 weeks. The final grade was determined by the mean of the two grades. 20 patients recruited in this study from November, 2022 to March, 2023 were analyzed.

**Table 2:** Sugaya Grade.

Grade	Description
I	sufficient thickness compared to normal cuff with homogeneous low signal
II	sufficient thickness compared to normal cuff with partial high‐intensity area
III	<50% thickness compared to normal cuff without discontinuity suggests partial‐thickness delamination tear
IV	minor discontinuity (1–2 slices) on both oblique coronal and oblique sagittal images suggests small full‐thickness tear
V	major discontinuity (>2 slices) on both oblique coronal and sagittal images suggests medium or large full‐thickness tear

### SNQ Analysis

In SNQ analysis, the ROI were defined as the RC TBI and tendon part of RC. An identical size of a 5 mm circle was applied to measure the signal intensity (SI). The tendon part was defined at the area 10 mm from the medial edge of RC enthesis. The area above the RC enthesis was considered the TBI area (including the interface and part of the distal tendon). (Figure 7C) SNQ was calculated by the following equation: SI/ background SI.

### Statistical Analysis

One‐way ANOVA was used for statistical analysis of experimental data. Graphical analysis was performed using Prism Graph 9 and Origin Pro (Student Version). Transcriptional analysis graphs were created using online platforms for data analysis and visualization. All data will be presented as mean ± standard deviation (Mean ± SD). Results with P < 0.05 (^*^) will be considered as statistical significance. For non‐parametric data, a Kruskal‐Wallis test was used for one‐way analysis of variance.

## Conflict of Interest

The authors declare no conflict of interest.

## Supporting information

Supporting Information 1

Supporting Information 2

## Data Availability

The data that support the findings of this study are available from the corresponding author upon reasonable request.
