# Supplementary material for: Bioactive Patch for Rotator Cuff Repairing via Enhancing Tendon‐to‐Bone Healing: A Large Animal Study and Short‐Term Outcome of a Clinical Trial
Source: Adv Sci (Weinh). 2024 Jun 23;11(31):2308443. doi: 10.1002/advs.202308443 (PMC11336973; doi:10.1002/advs.202308443)
Supplement: Supplementary file 1 — Supporting Information 1 [file ADVS-11-2308443-s001.docx]

Supporting Information

Bioactive patch for rotator cuff repairing via enhancing tendon-to-bone healing: a large animal study and short-term outcome of a clinical trial

*Yuhao Kang, Liren Wang, Shihao Zhang, Bowen Liu, Haihan Gao, Haocheng Jin, Lan Xiao, Guoyang Zhang, Yulin Li^*^, Jia Jiang^*^，Jinzhong Zhao^*^*

Figure S1 Top 30 coverage proteins detected in spectrometry analysis.

Figure S2 Release profile of Collagen-VI and TGFBI in vitro (28 Days).


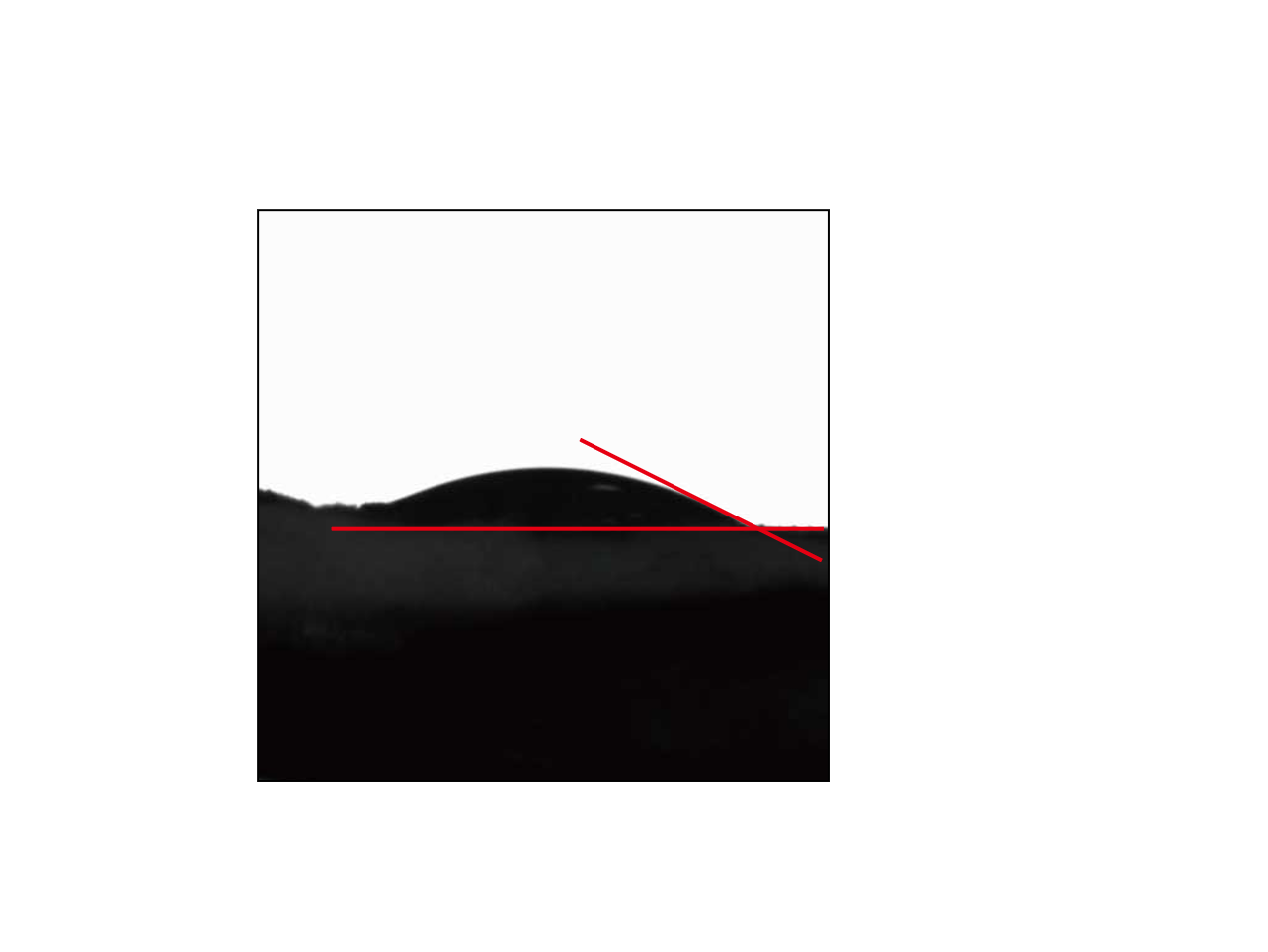


Figure S3 Representative figure of contact angle of UC scaffold.

Figure S4 UC extracts promotes cell proliferation. UC, Umbilical cord; Ctrl, Control. * P<.05 compared to control group


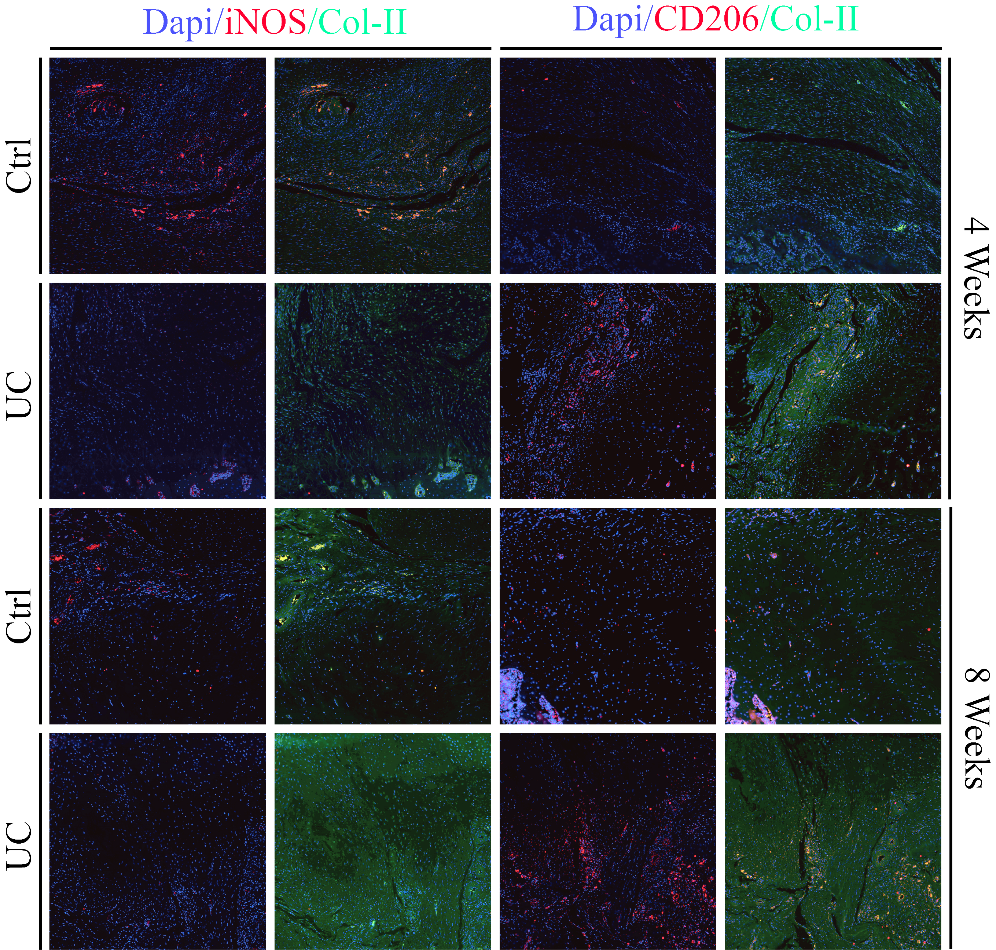


Figure S5 In vivo evaluation of Machrophage polarization in rat rotator cuff repair model.

After anesthetizing the rats with 1.5% sodium pentobarbital, the forelimbs of the rats were externally rotated, and a 2cm skin incision was made on the surface skin of the deltoid muscle. The deltoid muscle was split to expose the supraspinatus tendon, and the supraspinatus tendon was excised at its footprint on the greater tubercle. After scraping off the residual cartilage layer at the footprint area, a bone tunnel was created using a 22G needle, and the supraspinatus muscle was sutured back to its insertion point using No. 4-0 Prolene suture, followed by layer-by-layer suturing. The same procedure was performed on the opposite shoulder, and an umbilical cord scaffold (3*3mm) was placed between the tendon and bone before proceeding with layer-by-layer suturing, following the same method as in large animal models. Four and eight weeks postoperatively, DAPI/COL-II (Affinity, AF0135)/iNOS (Abclonal, A0312) and DAPI/COL-II/CD206 (Abclonal, A25433) immunofluorescence staining were performed to evaluate the immunomodulatory effects. The results suggest that at 4 weeks post-surgery, the fluorescence intensity of Collagen-II was low in both groups, with more iNOS+ cells in the control group than in the UC group, and fewer CD206+ cells than in the UC group. At 8 weeks post-surgery, the fluorescence intensity of Collagen-II increased, indicating cartilage layer regeneration. In the control group, the number of iNOS+ cells decreased compared to earlier, and the number of CD206+ cells further increased but was still less than in the UC group, indicating the UC scaffold possessing immunoregulation ability in vivo.


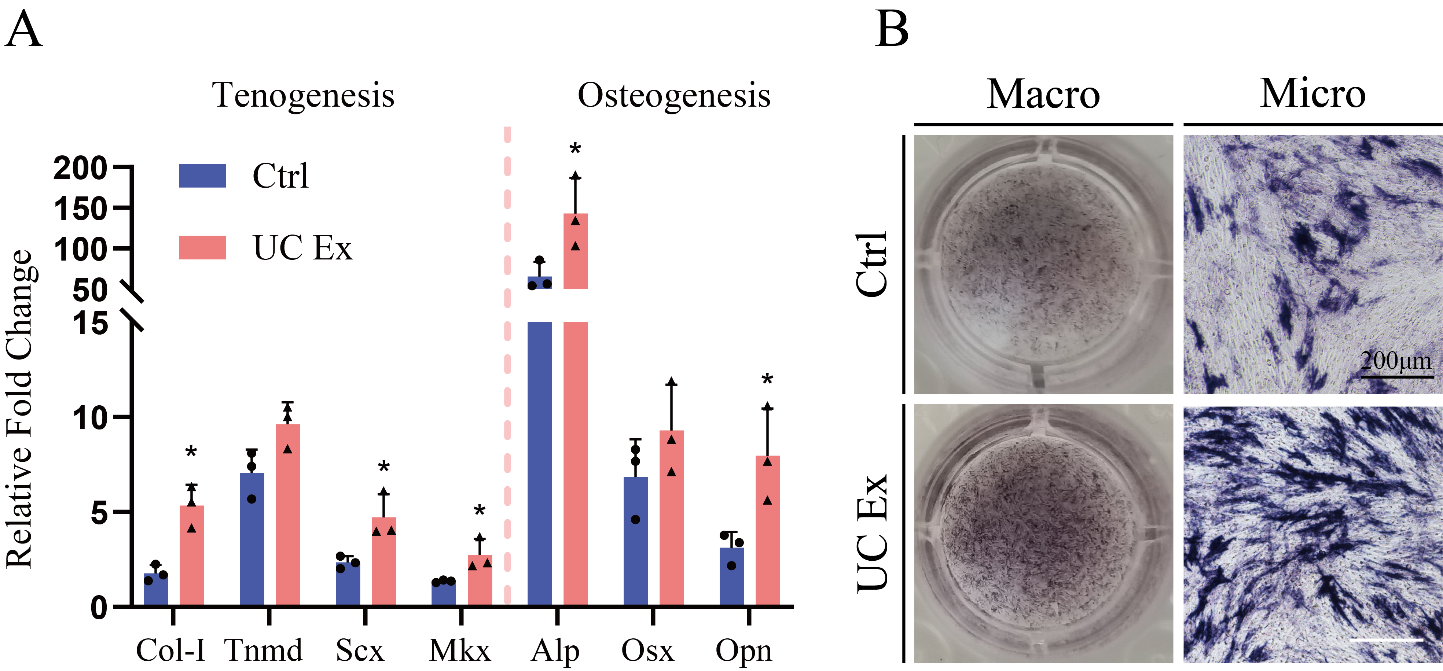


Figure S6 Tenogenesis and Osteogenesis evaluation.

BMSCs were induced for tenogenic differentiation for 7 days with DMEM or UC extracts complete medium containing 50μg/ml ascorbic acid, while the medium was supplemented with 100nM dexamethasone, 50 μM ascorbic acid and 10 mM β-glycerophosphate in osteogenic induction. The expression of tenogenic genes (Tnmd, COL1A1, Mkx and Scx) and osteogenic genes (Opn, Osx and Alp) were analyzed using RT-qPCR following standard protocol. The primer sequences are presented in Table S1. The results suggest that UC Extracts can promote the expression of tenogenesis-related genes such as COL-I, Mkx, and Scx. (P<0.05). However, there is no significant difference in Tnmd compared to the control group (P=0.06). Osteogenesis-related genes such as Alp and OPN also showed a significant increase (P<0.05), while there was no significant difference in Osx compared to the control group (P=0.24). ALP staining was also conducted for osteogenic evaluation (Beyotime, C3206), which indicated that UC extracts possessed osteogenic ability.


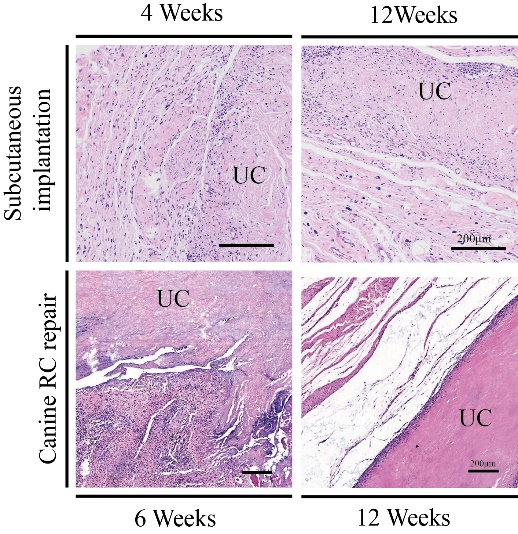


Figure S7 Biodegradable evaluation of UC scaffold in subcutaneous implantation rat model and canine RC repair model

Rats were anesthetized with 1.5% sodium pentobarbital and shaved, followed by routine disinfection. Subcutaneous implantation (1cm*1cm UC scaffold) was performed about 2 cm from the midline of the rat's spine. At 4- and 12-weeks post-implantation, the implants and surrounding tissues were excised for HE staining analysis. In the rat subcutaneous implantation model, partial degradation of the UC scaffolds was observed at 4 weeks post-implantation, with the degree of degradation increasing over time and no significant inflammatory response detected. In the canine rotator cuff repair model, scaffold residues were visible at 6 weeks post-surgery, with further degradation observed at 12 weeks, without significant inflammation.

| Species | Name | Sequence 5‘-3’ |
| --- | --- | --- |
| Human | GADPH | AGA AAA ACC TGC CAA ATA TGA TGA |
|  |  | TGG GTG TCG CTG TTG AAG TC |
|  | KDR | GAC TGA ATG CGG GAG GT |
|  |  | GGT GAG AGT GGG TTG GG |
|  | NOS | CGA GCT GGT CCT TGA GGT |
|  |  | TGA GGG TCA CAC AGG TTC C |
|  | VEGFA | GGA ACA CCG ACA AAC CC |
|  |  | AAT CCC CAA AGC ACA GC |
|  | PECAM1 | TTA TGA ACC TGC CCT GCT |
|  |  | TCA ACA GCC CCT CTG TAT C |
|  | COL2A1 | GGC AAT AGC AGG TTC ACG TAC A |
|  |  | CGA TAA CAG TCT TGC CCC ACT T |
|  | Aggrecan | TCG AGG ACA GCG AGG CC |
|  |  | TCG AGG GTG TAG CGT GTA GAG A |
|  | SOX-9 | GGC AAT AGC AGG TTC ACG TAC A |
|  |  | CGA TAA CAG TCT TGC CCA CTT |
|  | COL-1A1 | TCT GAA ACC CTC CTC CAC A |
|  |  | GCT TCC TCC CAC CCC TA |
|  | Tnmd | AAA TCG CAG CAC AGG AGT |
|  |  | TGA GTC GGC TAA CAG ATG C |
|  | Scx | AAC ACC CAG CCC AAA CAG A |
|  |  | GCC ACC TCC TAA CTG CGA A |
|  | Mkx | CAG AAT GGC GGG AAG GTG A |
|  |  | TTA AAT CTG GCT GTC GAA CGG TAT |
|  | Opn | CCG TGG GAA GGA CAG TT |
|  |  | TTG CTC TCA TCA TTG GCT T |
|  | Osx | GCA AAG CAG GCA CAA AGA |
|  |  | CAG GTG AAA GGA GCC CAT |
|  | Alp | TCC TGG GAG ACA AAG CAA T |
|  |  | GGC GCA TAG ATA AGG GGT T |
| Mouse | GADPH | CCT CGT CCC GTA GAC AAA ATG |
|  |  | TGA GGT CAA TGA AGG GGT CGT |
|  | CD80 | GAC CCT CCT GAT AGC AAG AA CAC |
|  |  | CGA AGG TAA GGC TGT TGT TTG TT |
|  | INOS | CAA CAG GAA CCT ACC AGC TCA CT |
|  |  | AGC CTG AAG TCA TGT TTG CCG |
|  | IL-1β | AGG CTC CGA GAT GAA CAA CAA A |
|  |  | GTG CCG TCT TTC ATT ACA CAG GA |
|  | Arg-1 | GGT GGC AGA GGT CCA GAA GAA |
|  |  | CCC ATG CAG ATT CCC AGA GC |
|  | IL-10 | AAT AAG CTC CAA GAC CAA GGT GT |
|  |  | CAT CAT GTA TGC TTC TAT GCA GTT G |

Table S1 RT-qPCR Primers

| N=5 | Ultimate load | Stiffness | Tensile strength | Strain | Young's Module |
| --- | --- | --- | --- | --- | --- |
| Mean | 15.70 | 6.43 | 7.85 | 0.24 | 32.14 |
| Std | 1.37 | 0.39 | 0.69 | 0.02 | 1.94 |
|  | N | N/mm | Mpa | % | Mpa |

Table S2 Mechanical characrization of UC scaffold.

|  | 6 Weeks | | | | | 12 Weeks | | | | | 24 Weeks | | | | |
| --- | --- | --- | --- | --- | --- | --- | --- | --- | --- | --- | --- | --- | --- | --- | --- |
|  | Ctrl | | UC | | P value | Ctrl | | UC | | P value | Ctrl | | UC | | P value |
|  | Mean | Std | Mean | Std |  | Mean | Std | Mean | Std |  | Mean | Std | Mean | Std |  |
| Cellularity | 1.3 | 0.5 | 1.8 | 0.7 | 0.21 | 2.3 | 0.5 | 2.8 | 0.4 | 0.09 | 2.5 | 0.5 | 3.2 | 0.4 | 0.04* |
| Proportion of cells resembling tenocytes | 1.5 | 0.5 | 1.8 | 0.7 | 0.40 | 2.0 | 0.0 | 2.3 | 0.5 | 0.14 | 1.8 | 0.4 | 2.8 | 0.7 | 0.02* |
| Proportion of cells oriented parallel | 1.7 | 0.5 | 2.3 | 0.7 | 0.12 | 2.2 | 0.4 | 3.2 | 0.7 | 0.02* | 2.2 | 0.4 | 3.5 | 0.5 | <0.01* |
| Vascularity | 2.7 | 0.9 | 3.2 | 0.7 | 0.36 | 2.3 | 0.5 | 3.8 | 0.4 | <0.01* | 2.3 | 0.5 | 3.7 | 0.5 | <0.01* |
| Proportion of fibers of large diameter | 1.2 | 0.4 | 1.5 | 0.5 | 0.26 | 2.0 | 0.0 | 2.0 | 0.0 | - | 2.3 | 0.5 | 3.2 | 0.7 | 0.05 |
| Proportion of fibers oriented parallel | 1.2 | 0.4 | 2.2 | 0.7 | 0.02* | 2.5 | 0.8 | 2.8 | 0.4 | 0.40 | 2.2 | 0.4 | 3.5 | 0.5 | <0.01* |
| Remodeling of tendon-to-bone insertion | 2.5 | 0.5 | 2.2 | 0.9 | 0.48 | 2.7 | 0.5 | 3.0 | 0.0 | 0.14 | 2.3 | 0.5 | 3.8 | 0.4 | <0.01* |

Table S3 Modified tendon enthesis maturing score. UC, Umbilical cord; Ctrl, Control. * P<.05 compared to control group

| **Classification** | | Meaning of score value | | | | |
| --- | --- | --- | --- | --- | --- | --- |
|  |  | 0 | 1 | 2 | 3 | 4 |
| **Cell type/**  **response** | **Polymorphonuclear cells** | 0 | Rare, 1 to 5/phf^a^ | 5 to 10/phf | Heavy infiltrate | Packed |
|  | **Lymphocytes** | 0 |  |  |  |  |
|  | **Plasma cells** | 0 |  |  |  |  |
|  | **Macrophages** | 0 |  |  |  |  |
|  | **Giant cells** | 0 | Rare, 1 to 2/phf | 3 to 5/phf |  | Sheets |
|  | **Necrosis** | 0 | Minimal | Mild | Moderate | Severe |
| **Fibrosis** | | 0 | Narrow | Moderately thick band | Thick band | Extensive band |
| **Neovascularization** | | 0 | Minimal capillary proliferation, focal, 1 to 3 buds | Groups of 4 to 7 capillaries with supporting fibroblastic structures | Broad band of capillaries with supporting fibroblastic structures | Extensive band of capillaries with supporting fibroblastic structures |
| **Degradation** | | 0 | Minimal degradation of implant, some minor dissolution on edges, cracks in implant and/or small fragment present | Moderate degradation of implant with cracks in implant and/or some fragments | Marked degradation of implant with presence of several fragments | Abundant degradation of implant with (almost) complete fragmentation |
| **Phagocytosis** | | 0 | Minimal phagocytosis, some cells present with phagocytized material | Moderate phagocytosis, several cells and/or groups of cells with phagocytosis | Marked phagocytosis, several groups of cells with phagocytosis and/or zone of phagocytic cells around implant | Severe phagocytosis, all material phagocytized either as large fragments or as small parts in individual macrophages |

Table S4. Semiquantitative evaluation of the tissue reaction after scaffold implantation.

|  | | | | | | | | | | | | | | | |
| --- | --- | --- | --- | --- | --- | --- | --- | --- | --- | --- | --- | --- | --- | --- | --- |
|  | 6 Weeks | | | | | 12 Weeks | | | | | 24 Weeks | | | | |
|  | Ctrl | | UC | | P value | Ctrl | | UC | | P value | Ctrl | | UC | | P value |
| Cell type/response | Mean | Std | Mean | Std |  | Mean | Std | Mean | Std |  | Mean | Std | Mean | Std |  |
| Polymorphonuclear cells | 0.7 | 0.7 | 0.7 | 0.5 | 1.00 | 0.0 | 0.0 | 0.0 | 0.0 | - | 0.0 | 0.0 | 0.0 | 0.0 | - |
| Lymphocytes | 0.7 | 0.5 | 0.5 | 0.5 | 0.60 | 1.0 | 0.0 | 0.8 | 0.4 | 0.34 | 0.8 | 0.4 | 0.7 | 0.5 | 0.55 |
| Plasma cells | 0.2 | 0.4 | 0.7 | 0.5 | 0.09 | 0.0 | 0.0 | 0.3 | 0.7 | 0.34 | 0.0 | 0.0 | 0.0 | 0.0 | - |
| Macrophages | 1.5 | 0.5 | 1.3 | 0.5 | 0.60 | 1.0 | 0.0 | 1.2 | 0.4 | 0.34 | 1.0 | 0.0 | 1.0 | 0.0 | - |
| Giant cells | 0.0 | 0.0 | 0.0 | 0.0 | - | 0.2 | 0.4 | 0.0 | 0.0 | 0.34 | 0.5 | 0.5 | 0.0 | 0.0 | 0.05 |
| Necrosis | 0.3 | 0.5 | 0.3 | 0.5 | 1.00 | 0.0 | 0.0 | 0.0 | 0.0 | - | 0.0 | 0.0 | 0.0 | 0.0 | - |
| Fibrosis | 1.0 | 0.0 | 1.0 | 0.0 | 1.00 | 1.0 | 0.0 | 1.0 | 0.0 | - | 1.0 | 0.0 | 1.0 | 0.0 | - |
| Neovascularization | 0.5 | 0.5 | 0.7 | 0.5 | 0.60 | 0.0 | 0.0 | 0.2 | 0.4 | 0.34 | 0.3 | 0.5 | 0.5 | 0.5 | 0.60 |
| Phagocytosis | 0.3 | 0.5 | 0.3 | 0.5 | 1.00 | 0.5 | 0.5 | 0.2 | 0.4 | 0.26 | 0.2 | 0.4 | 0.0 | 0.0 | 0.34 |
| Total | 8.5 | 2.6 | 9.0 | 3.2 | 0.79 | 5.8 | 0.7 | 6.0 | 2.4 | 0.89 | 6.2 | 1.9 | 4.8 | 1.1 | 0.20 |

Table S5. Tissue response after UC scaffold implantation. UC, Umbilical cord; Ctrl, Control.
